# Culture independent analysis using *gnd* as a target gene to assess *Escherichia coli* diversity and community structure

**DOI:** 10.1038/s41598-017-00890-6

**Published:** 2017-04-12

**Authors:** Adrian L. Cookson, Patrick J. Biggs, Jonathan C. Marshall, Angela Reynolds, Rose M. Collis, Nigel P. French, Gale Brightwell

**Affiliations:** 1 AgResearch Limited, Hopkirk Research Institute, Palmerston North, New Zealand; 2 mEpiLab, Hopkirk Research Institute, Massey University, Palmerston North, New Zealand; 3 Massey Genome Service, New Zealand Genomics Limited, Massey University, Palmerston North, New Zealand; 4 Institute of Fundamental Sciences, Massey University, Palmerston North, New Zealand

## Abstract

Current culture methods to investigate changes in *Escherichia coli* community structure are often slow and laborious. Genes such as *gnd* (6-phosphogluconate dehydrogenase) have a highly variable nucleotide sequence and may provide a target for *E*. *coli* microbiome analysis using culture-independent methods. Metabarcoded PCR primers were used to generate separate libraries from calf faecal samples for high throughput sequencing. Although a total of 348 separate *gnd* sequence types (gSTs) were identified, 188 were likely to be due to sequencing errors. Of the remaining 160 gSTs, 92 did not match those in a database of 319 separate *gnd* sequences. ‘Animal’ was the main determinant of *E*. *coli* diversity with limited impact of sample type or DNA extraction method on intra-host *E*. *coli* community variation from faeces and recto-anal mucosal swab samples. This culture-independent study has addressed the difficulties of quantifying bacterial intra-species diversity and revealed that, whilst individual animals may harbour >50 separate *E*. *coli* strains, communities are dominated by <10 strains alongside a large pool of subdominant strains present at low abundances. This method will be useful for characterising the diversity and population structure of *E*. *coli* in experimental studies designed to assess the impact of interventions on the gut microbiome.

## Introduction

*Escherichia coli* has been extensively studied as a model organism and is the predominant facultative anaerobe in the gastro-intestinal tract of mammals^1^. It is a commensal associated with the gut mucosa^2^ and also a significant intestinal and extra-intestinal pathogen of veterinary and public health concern^3^. Using cultures purified from complex faecal or environmental samples, whole genome sequence and multi-locus sequence typing (MLST) data has provided new insights into taxonomic diversity of cultured representatives of *E*. *coli* phylotypes and closely related clades^4–10^. Other studies have utilised substrate biotyping, serological or molecular subtyping of isolates cultured from faeces to examine temporal changes in *E*. *coli* community structure in humans or cattle fed contrasting diets^11–15^. High throughput sequencing of barcoded amplicon libraries is an appealing alternative to the use of culture-based methods for examining microbial diversity. Such methods targeting the 16S rRNA gene are well-developed for establishing the microbial diversity of complex environmental or clinical samples. However the 16S rRNA gene is not amenable to the study of intra-species variation with microbial community structure often described at the genus or family level. Indeed the *E*. *coli* 16S rRNA gene is indistinguishable from that of *Shigella* species which belong to the same species complex^16, 17^.

*E*. *coli* are highly diverse at the genomic level, but the genetic structure has permitted phylogenetic separation of human strains into at least six different phylogroups (A, B1, B2, D, E and F) using PCR-based methods targetting *chu*A, *yja*A and TspE4.C2 alleles^8, 18, 19^. Other cryptic *E*. *coli* strains phenotypically indistinguishable from *E*. *coli* and more abundant in animal faeces have been identified by multi-locus sequence typing^20^, clade-specific single nucleotide polymorphisms (SNPs) present in *chu*A and *aes* ^5^ and genome sequencing^6^. Using these approaches the presence of five sub-species within *E*. *coli* has been suggested^21^. Culture-independent real time PCR methods have targetted alleles matching the four major *E*. *coli* phylogroups (A, B1, B2 and D) in human faeces. Their relative proportions revealed high within-individual diversity including the presence of minor clones potentially associated with temporal variation of the *E*. *coli* microbiota within individuals^22^. More recently shotgun metagenomic methods for distinguishing the *E*. *coli* microbiota have achieved microbial resolution at the strain-level but may be limited in their resolution in complex samples containing *E*. *coli* strains at low abundance^23–26^.

*E*. *coli* evolution and population structure is more heavily influenced by recombination than mutation^27^ and certain regions of the *E*. *coli* chromosome, such as the O-antigen biosynthesis gene cluster (O-AGC, *rfb* operon), are predisposed to horizontal gene transfer and recombination events associated with purifying selection^28^. To date, serological reagents raised against the O-antigen polysaccharide, the outermost part of the lipopolysaccharide (LPS), are available for 184 separate serogroups allowing subtyping of *E*. *coli* strains for both outbreak investigations and general surveillance. The O–AGC and adjacent loci are prone to genetic rearrangement with many defined and well-characterised O-antigen somatic serogroups recognised^29^, however the isolation of untypable *E*. *coli* strains that do not cross-react with available O-antigen antisera is common^30^.

The presence of certain *E*. *coli* genotypes/phenotypes in low numbers in a complex sample may lead to difficulties in their detection and isolation using routine culture media. Previous studies characterising polymorphisms in *gnd* ^31–36^ (the gene encoding 6-phosphogluconate dehydrogenase) and its location close to the O-AGC, a region of high recombination associated with the *E*. *coli* chromosome^28^, have seen it described as a passive hitch-hiker of recombination events that determine both LPS antigenic changes and diversifying selection^32^. 6-phosphogluconate dehydrogenase is the third enzyme of the pentose phosphate pathway but the extent to which nucleotide polymorphisms impact NAD^+^ binding and phosphogluconate dehydrogenase activity of the enzyme has not been fully investigated^32, 37^. The increasing availability of whole genome sequence (WGS) data from *E*. *coli* isolates now provides a valuable resource to target polymorphic genes as candidates for measuring intra-species diversity^38^, and thus, this work aimed to target *gnd* as a candidate to measure intestinal *E*. *coli* diversity in parallel with the development of a *gnd* database, for cross-referencing purposes. The notion that a respective *gnd* sequence could be related to an adjacent O-antigen biosynthesis gene cluster has been explored previously^33, 34^, however *gnd* polymorphisms precluded the amplification and sequencing of some loci^34^. Therefore we investigated whether the *gnd* locus was a suitable target for culture-independent studies to assess *E*. *coli* diversity and developed a method for evaluating and correcting for biases introduced by sequencing error. Such an approach will be valuable for accurately and reliably assessing changes in the gut microbiome in response to challenges such as dietary interventions and the use of antimicrobials. Understanding the relative contribution of factors, such as the animal host, sample type and extraction method, will also help in the design of experimental studies.

## Results

### Construction of a *gnd* database

Using *E*. *coli* sequence data, a database of 240 *gnd* sequence types (gSTs) was constructed containing representative 284 bp *gnd* sequences from all 184 recognised *E*. *coli* O serogroups (Supplementary Table 1), 28 gSTs from *E*. *coli* described as untypable or ‘rough’, and three new, as yet undesignated O serogroups^30^. Seventy of 184 *E*. *coli* O serogroups were represented by a single unique gST, 113 O serogroups were either represented by more than one gST or a gST that was common to more than one serogroup and the final O serogroup (O165) contained two *gnd* alleles each characterised by a separate gST. The degenerate primers also amplified distinct *gnd* alleles from other Enterobacteriaceae including *Escherichia fergusonii*, and species of the genera *Citrobacter*, *Serratia*, *Salmonella* and *Enterobacter*. The *gnd* sequences and associated O-AGC of *Shigella* are known to be serologically and genetically identical, or very similar, to some *E*. *coli* antigens^16^ but *Shigella* species were not included in the extended validation panel.

### Comparative analysis of bovine *E*. *coli* faecal communities

**Figure 1:**
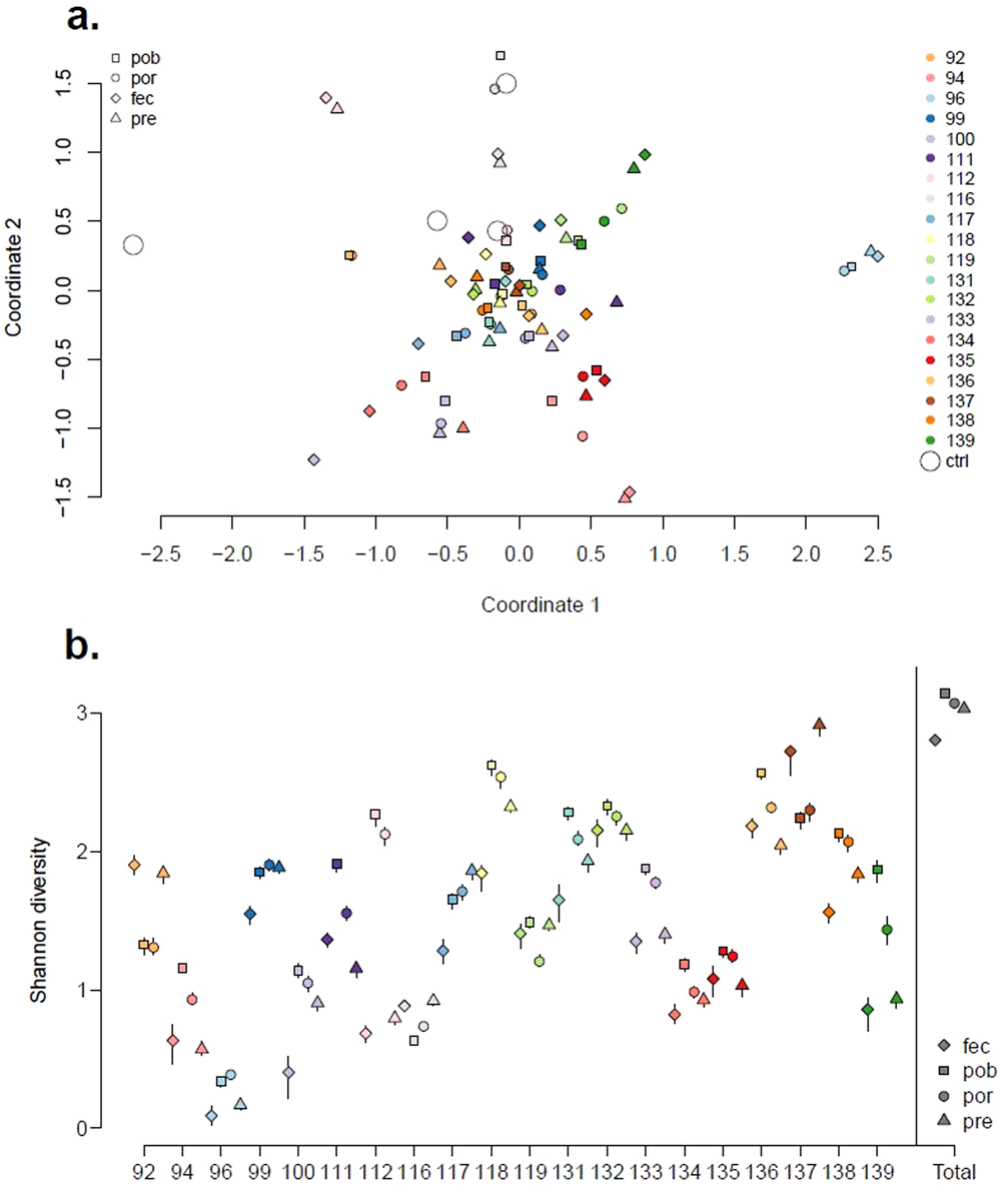
*gnd* sequence type (gST) clustering and diversity at the animal and library level. **(a**) Clustering of gSTs obtained from 20 calves and four control samples using multi-dimensional scaling plot to illustrate the gST similarity derived from each of the four extraction methods (pre-enrichment, ‘pre’; post-enrichment boiled lysate, ‘pob’; post-enrichment, spin-column, ‘por’ and faecal, ‘fec’) for each animal and 4 mock libraries. The MDS plot provides a visual representation of the distance matrix encompassing the similarity of gSTs found in each of the 84 libraries, i.e. four extraction methods (symbol shape) and 20 animals (symbol colour). Symbols close together indicate libraries containing similar gSTs; symbols far apart indicate libraries containing dissimilar gSTs. (**b**) Diversity of gSTs obtained from 20 calves using Shannon Index to illustrate the number of different gSTs and the variation in gST relative abundance obtained from each of the four extraction methods (pre-enrichment, ‘pre’; post-enrichment boiled lysate, ‘pob’; post-enrichment, spin-column, ‘por’ and faecal, ‘fec’) for each animal. Sequence data obtained from the four libraries originating from calf 96 contained between 3 and 13 different gSTs; in contrast sequence data obtained from the four libraries originating from calf 137 contained between 40 and 82 different gSTs. The total gST diversity across the 80 libraries is illustrated on the right of the x axis. The error bars represent 95% confidence intervals.

The four mock library datasets corresponding to eight separate quality threshold were analysed (Supplementary Fig. 1, Supplementary Table 2) and the MiSeq dataset with a read quality threshold of 1 base call error every 67 nucleotides (*P* = 0.015) was chosen as a test dataset for the development of a framework for the analysis of *E*. *coli* diversity. In total, the *P* = 0.015 threshold generated 183309 reads from the 92 sample libraries (Supplementary Table 3a). Further analysis was only undertaken on 80 sample libraries from 20 animals; sequence data from three animals (97, 98, and 120) were not included due to very low (<150) total read numbers associated with libraries generated with the *P* = 0.015 (Q18.2) threshold (Supplementary Table 3b). Multi-dimensional scaling analysis of the 80 sample libraries indicated that there was considerable clustering at the animal level (Fig. 1a). However the four libraries associated with animal 96 clustered away from the other libraries and were the least diverse (Fig. 1b).

Four separate libraries were made from each individual animal to determine whether *E*. *coli* communities varied between (i) RAMS and faecal samples, (ii) pre and post-enrichment of RAMS samples in mTSB, and (iii) post-enrichment RAMS samples where DNA was extracted using a crude boiled lysate or a spin-column method. Using multivariate analysis of variance to assess the proportion of the variation in relative gST abundances across libraries due to the extraction method, and between and within-calf variability, we were able to demonstrate that ‘Animal’ contributed most to variation (81.2%), compared to ‘residuals’ (17.6%) and ‘extraction method’ (fec, pre, pob, por) (1.2%) (Supplementary Table 4). Furthermore, there was no evidence to suggest that clustering at the library gST diversity level was influenced by treatment with the bifidobacterial preparation (*P* = 0.53).

A total of 348 gSTs with ≥10 reads were obtained from the 80 sample libraries at the *P* = 0.015 quality threshold (Supplementary Table 3). All gSTs with less than 10 reads across the 80 sample libraries dataset at the *P* = 0.015 quality threshold were discarded. To assist with gST designation of output datasets, if a gST was identical with a database gST encompassing multiple O serogroups, the gST was categorised with the first numerical serogroup e.g. the O153A gST also matches the sequence from O156 and ONTQ (Supplementary Table 2). Hierarchical clustering (Supplementary Fig. 2) indicated that many clusters were identified as being composed of a high abundance parent gST, often matching a gST from the database, that clustered with less abundant novel gSTs that differed from the parent by one SNP. These SNP variants were described as daughter SNP types and increased linearly with the relative abundance of the parent *gnd* type (Supplementary Fig. 2, inset). CD-HIT was used to group the daughter SNP types with a related parent, using cluster analysis of the 348 gSTs from the *P* = 0.015 dataset at the 99.6% identity level, i.e. at the level of a single SNP, and 148 gSTs including 62 matching with the *gnd* database and 86 novel gSTs were identified. However, 7 gSTs matching database (cultured) entries clustered with other gSTs that differed by one SNP (Supplementary Table 5) suggesting a potential limitation of simple clustering methods. E.g. the O176 gST (6426 reads) clustered with the O17 gST (9425 reads).

### Application of the Error Correction model and clustering at single SNP level to assess *E*. *coli* diversity

**Figure 2:**
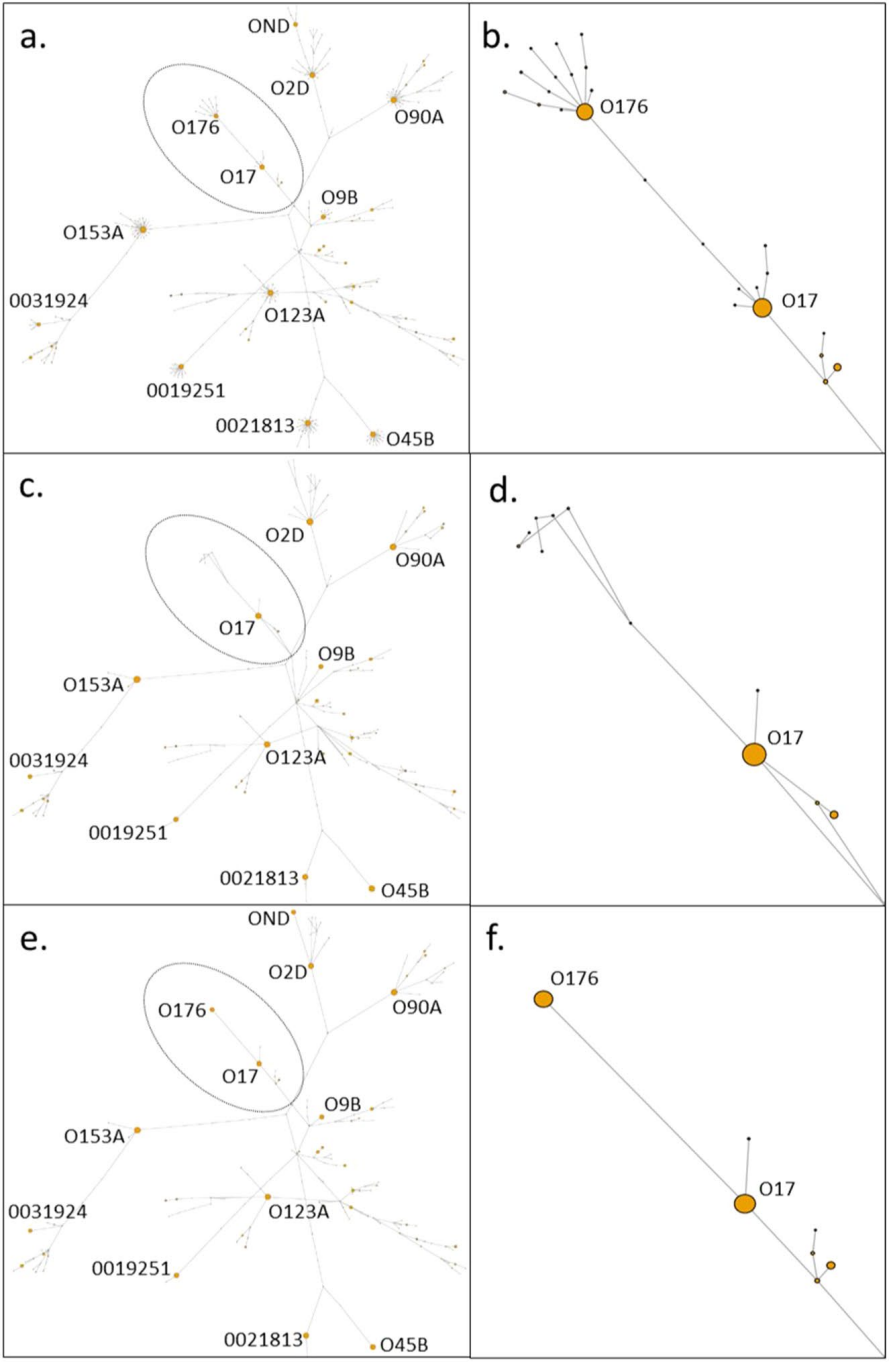
Effect of clustering using CD-HIT and EC model on relative abundance and total number of gSTs. To illustrate the discordance between clustering and statistical modelling methods in determining the genetic diversity and abundances of all 348 gSTs generated from the *P* = 0.015 dataset, a minimum spanning tree was generated with highly abundant gST (>4000 reads) nodes labelled. Node size indicates relative abundance of gST reads in the *P* = 0.015 dataset. (**a)** Unadjusted minimum spanning tree of all 348 gSTs generated from the *P* = 0.015 dataset with branch containing O17 (9432 reads) and O176 (6527 reads) gST nodes that differ by a one SNP, labelled with dotted black oval. (**b**) Enlarged branch of unadjusted minimum spanning tree containing O17/O176 gST nodes, illustrating low abundance daughter gSTs (smaller nodes) that differ from O17 and O176 nodes by one SNP. (**c**) Minimum spanning tree of gSTs generated from the *P* = 0.015 dataset clustered at 99.6% similarity level (one SNP) using CD-HIT. Branch containing O17 gST (16132 reads) labelled with dotted black oval. (**d**) Enlarged branch of minimum spanning tree containing O17 gST node, illustrating the absence of the O176 gST node having been clustered with with O17 gST due to 99.6% similarity (one SNP). (**e**) Minimum spanning tree of gSTs generated from the *P* = 0.015 dataset following application of the EC model with branch containing O17/O176 gST nodes that differ by a one SNP, labelled with dotted black oval. (**f**) Enlarged branch of minimum spanning tree containing O17 (9610 reads) and O176 (6536 reads) gST nodes, illustrating the absence of low abundance daughter gST nodes through clustering with highly abundant O17 and O176 nodes. O176 gST node not clustered with O17 gST node using EC model despite 99.6% similarity (one SNP difference) as highly abundant.

The application of the Error Correction (EC) model on the *P* = 0.015 test dataset resulted in 188 gSTs being removed, and the identification of 160 gSTs, of which 92 were novel and 68 matched the database (Supplementary Table 6). Almost all (187/188) of the discarded gSTs were novel with only the O25A gST matching a database sequence. BLAST analysis was performed on the 92 novel gSTs from the *P* = 0.015 dataset and indicated a best match for *E*. *coli*, *Escherichia fergusonii* or *Shigella* spp. for almost all (91 of 92) sequences (Supplementary Table 7). The outstanding gST matched *Klebsiella pneumoniae*. Compared to clustering at the single SNP level using CD-HIT, fewer gSTs were removed with the EC model. Particular gSTs may have be retained with the EC model as they differed from the parent gST by a single SNP, and they were present at a relatively high abundance so were unlikely to be associated with sequencing error. Alternatively, low abundance gSTs were retained with the EC model because the gST with the most similar nucleotide sequence was not abundant enough to produce the required sequencing errors, given the estimated error rate. To illustrate the effect of the EC model and to compare it to clustering using CD-HIT, a minimum spanning tree was used to visualise the genetic diversity and abundances of the uncorrected and corrected data (Fig. 2) where distances between gSTs were again measured by the number of different bases (Fig. 2a and b). Thus the O17 and O176 gSTs that differ by one SNP and were clustered together using CD-HIT (Fig. 2c and d), but were separated using the EC model due to their high relative abundance (Fig. 2e and f).

SNPs associated with likely error and novel gSTs were mapped against their most similar parent gST and closest matching gST from database respectively (Supplementary Fig. 3a and b). Sixty one of the 188 gSTs removed by the EC model were associated with a single non-synonymous amino acid change (Supplementary Fig. 4a). When the 92 novel gSTs identified using the EC model were compared with gSTs from the *gnd* database to identify SNPs (Supplementary Fig. 3b), common SNPs were often a characteristic of novel gSTs that shared a most similar parent. Non-synonymous changes were associated with 29 of the 92 novel gSTs (Supplementary Fig. 4b). SNPs were also mapped against the 3 codon positions of novel gSTs and gSTs removed using the EC model (Supplementary Fig. 5).

**Figure 3:**
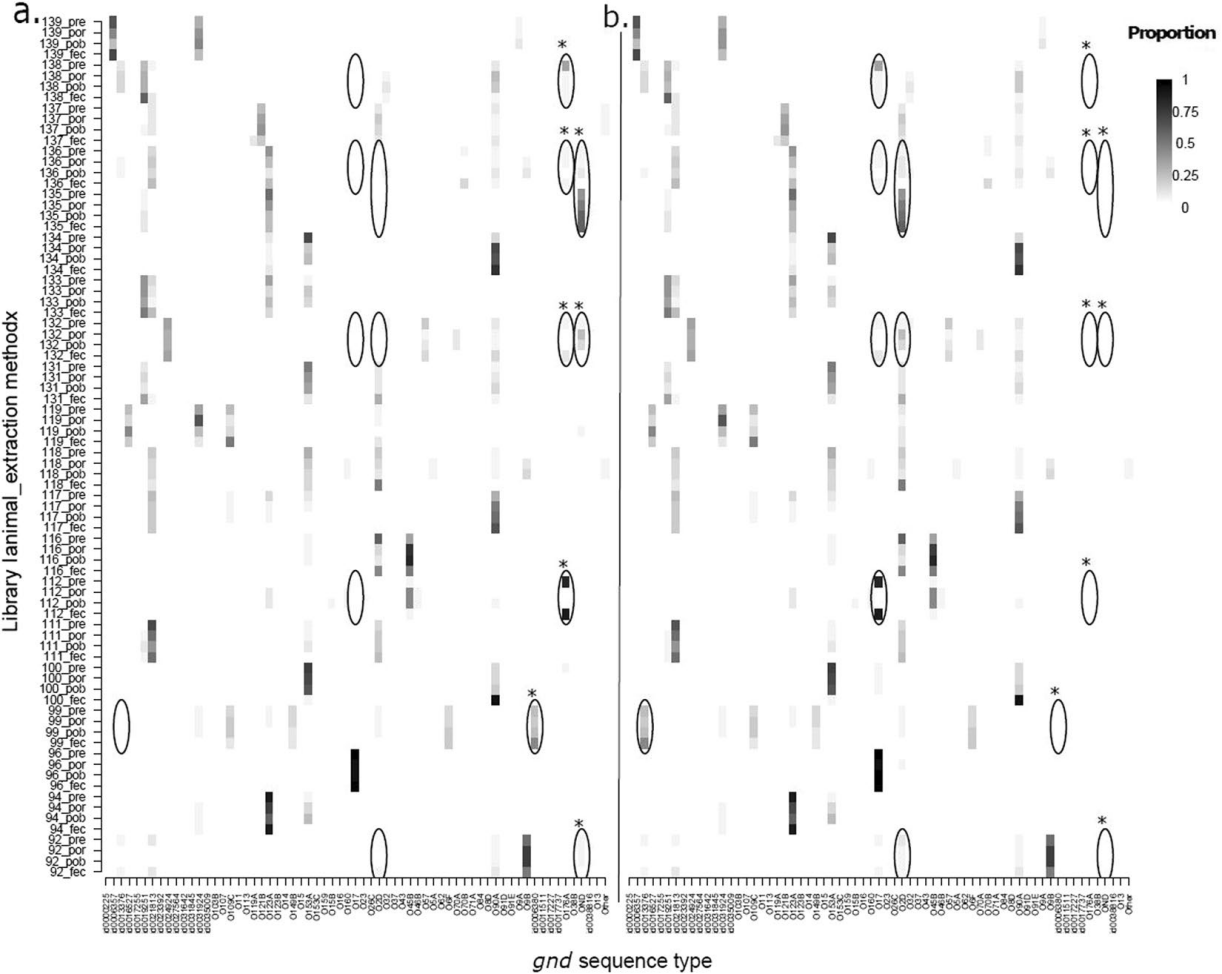
*E*. *coli* communities detected from calf faeces. Heat map to illustrate the relative proportions of gSTs along x axes (where present in sample library at >1% abundance) determined from amplicon libraries (n = 80) generated from template DNA prepared using four extraction methods (pre-enrichment, ‘pre’; post-enrichment, boiled lysate, ‘pob’; post-enrichment, spin-column, ‘por’ and faecal, ‘fec’) for each of 20 animals, along y axes. Proportions of gSTs associated with each animal and extraction method analysed using (**a**) the EC model and (**b**) clustering at the 99.6% similarity level (one SNP difference) using CD-HIT. Both methods reveal limited impact of sample type or DNA extraction method on intra-host *E*. *coli* community variation from faeces and recto-anal mucosal swab samples. Black ellipses represent gSTs (e.g. O17 and O176, or OND and O2D) that differ by one SNP. gSTs marked with an asterisk using the EC model (**a**) are combined with other gSTs after cluster analysis at the 99.6% similarity level (one SNP) (**b**). Squares of increasing shading represent gSTs at proportions from 1% (very pale) to 100% (black) associated with individual animal and extraction method, i.e. 80 libraries.

When the relative abundance of each gST was adjusted using the EC model and used to determine the relative proportion according to total reads per library, relatively few gSTs dominated the overall *E*. *coli* community at a level >1.0% (Fig. 3) with there being a vast pool of less abundant strains at the <1.0% level (Supplementary Table 8). All four libraries associated with animal 116 were dominated by three gSTs (O45B, O2D and O153A) with a combined relative proportion of >95% of all *gnd* sequence reads (Fig. 3, Supplementary Table 8). Animal 137 however was dominated by between 9 and 17 gSTs at a level of >1.0% (Fig. 3, Supplementary Table 8).

### Comparison of culture-based identification of gSTs and high throughput genome sequencing methods

If present at a high relative abundance post-enrichment, most individual *E*. *coli* strains are comparatively easy to isolate on selective agar media. Therefore to provide an initial comparison of gSTs obtained from culture-independent and culture dependent methods, Sanger sequencing of the *gnd* allele from cultured isolates was undertaken and compared to gSTs obtained from matching animals/libraries using high throughput sequencing methods. Seven pale non-lactose fermenting colonies and 98 purple lactose fermenting colonies were isolated from RAMS enrichments after incubation on MacConkey agar (Supplementary Table 9). A 2*gnd*F/2*gnd*R PCR product was obtained from 94 isolates; the 2*gnd*F and *GND*-R^39^ primer combination was used to generate an amplicon of 650 bp from 10 of the remaining 11 isolates. Thirty three isolates were confirmed as being concordant with the equivalent serogroup (Supplementary Table 9) using serogroup specific PCR primers^40^. No such concordance using prospective serogroup specific primers was obtained with 27 isolates (Supplementary Table 9). Interestingly, contrasting sequence data has been generated for the O153 O-AGC that may be associated with the failure of the specific PCR described for this serogroup^40, 41^.

There was strong concordance of gSTs obtained from cultured isolates and culture-independent data, however the gSTs from 6 isolates corresponded with equivalent libraries generated with slightly less stringent sequencing quality thresholds (*P* = 0.018 and *P* = 0.021) (Supplementary Table 9).

The 284 bp *gnd* amplicon from three isolates (AGR2693, AGR2717 and AGR2723) matched the novel gST id0018949. A GenBank BLAST search of this gST indicated a closest match to both *E*. *coli* and *K*. *pneumoniae* sequences (Supplementary Table 7). To provide an indication whether the isolates were *E*. *coli* or *K*. *pneumoniae* their growth on media containing citrate and urea was investigated.

Thirteen isolates were unable to be positively identified as *E*. *coli* as GenBank BLAST searches of their respective gSTs, obtained using Sanger sequencing, indicated a closest match to *E*. *coli* and/or *K*. *pneumoniae* sequences (Supplementary Tables 9 and 10). Unlike *K*. *pneumoniae*, most *E*. *coli* isolates are unable to metabolise citrate and urea^42^. Upon growth on media to demonstrate urea and citrate metabolism no growth was observed by nine of the thirteen isolates (Supplementary Table 9), suggestive of *E*. *coli*. The *gnd* allele from AGR2761 was closely related to that of an *E*. *coli* serogroup O8 strain (AB010150) that has LPS structural similarities with *Klebsiella* spp^9^. One isolate that was not amenable to amplification using the *gnd* primers used in this study was identified as *Providencia stuartii* using 16S rRNA gene sequencing (data not shown).

## Discussion

Gut *E*. *coli* exist in a symbiotic relationship with the anaerobic members of the gut microbiota that through the degradation of complex mucin polysaccharides, provide the mono- and di-saccharides *E*. *coli* require for growth^43^. Several recent studies have suggested a complex relationship between different *E*. *coli* phylogroups within the human and animal gut^20, 44^ identifying a pool of less abundant types dominated by others^22, 24^. Peturbations of the mucin-degrading members of the gut microbiota caused by environmental or dietary factors, or invading pathogenic *E*. *coli* may, in turn, influence changes in the *E*. *coli* microbiota permitting less abundant clones to become dominant.

In this work we explored the use of degenerate primers to amplify a segment of the *gnd* locus to provide an indication of *E*. *coli* diversity from cattle and the putative identification of low abundance *E*. *coli* types from multiple animals and samples. With an amplicon sequencing method we have succeeded in providing a much improved resolution of the faecal *E*. *coli* microbiota, including low abundance *E*. *coli* types, compared with culture methods and shotgun metagenomic studies described to date. Recent observations have noted that the O serogroups of contrasting immunogenicity are unable to be differentiated using genetic methods suggesting that gene regulation or O antigen synthesis methods contribute to antigenic hetergeneity^29, 45^. As a result some *E*. *coli* O serogroups of contrasting immunogenicity share the same O-AGC and cannnot be distuiguished using genetic methods^29, 45^. Diversity of the *gnd* allele influenced the amplification of a small number of *E*. *coli* isolates obtained in this study with gSTs most similar to *gnd* sequences associated with *Klebsiella* capsular polysaccharide synthesis (cps) gene clusters (Supplementary Table 10).

The *gnd* allele (1407 bp) is highly prone to recombination resulting in significant variation at the nucleotide level^32^. Previous studies have attempted to use generic PCR primers to amplify a portion of the *gnd* allele but nucleotide polymorphisms precluded the amplification of amplicons from some *E*. *coli* ^34^. The degenerate primers developed in this work target a relatively short part of the *gnd* allele (284 bp), and were designed to generate amplicons amenable for Illumina MiSeq platform, covering both domains of the 6-phosphogluconate dehydrogenase enzyme. Nevertheless, despite the amplification of only 20% of the allele, 160 gSTs were identified from 20 animals. Compared to previous culture-based studies^12, 13^, the observations stemming from this work have provided a much enhanced resolution of the bovine *E*. *coli* microbiota and offer a basis upon which further studies to examine factors determining flux of the *E*. *coli* microbiota within individual animals or humans. Further work may allow the improvement the *gnd* PCR primer sequences to enhance method sensitivity for recovery of contrasting gSTs as further *E*. *coli* genome sequence data becomes available. Furthermore, future sequencing platforms may provide additional options for longer *gnd* amplicons to be generated thereby permitting additional DNA polymorphisms outside of the current amplification region to further differentiate gSTs. Interestingly, several pairs of serogroup specific PCR primers (O9, O84, O91) did not confirm the identity of an isolate from which the respective gST (O9A, O84, O91D) was identified using Sanger sequencing. These data suggest that *gnd* sequence variability external to the 284 bp 2*gnd*F/2*gnd*R amplicon may be associated with novel O-antigens or that contrasting O-AGC DNA sequences are associated with antigenic similar O serogroups, as seen recently with O1 and O2 serogroups^46^.

Prior work with *E*. *coli* serogroup O157 has demonstrated that contrasting gSTs provide an indication of separate recombination or horizontal gene transfer events giving rise to separate pathotypes^35^. In contrast, the 284 bp gST associated with O26 strains appears to be stable across different pathotypes^47^ with a single O26:H6 isolate the only representative of a contrasting O26 gST^47^. The variation of gST within defined serogroups therefore provides some evidence of independent recombination or horizontal transfer events that have resulted in *E*. *coli* strains expressing structurally similar LPS antigens. Thus, recombination events associated with the transfer of O-AGC may result in novel gSTs but with no change of O-antigenicity.

Although on average 30 gSTs were associated with each library, some low abundance gSTs with a total count of <10 reads across our *P* = 0.015 test dataset, or present in datasets with a lower quality threshold, may have been overlooked using culture-independent methods. Relaxing the stringency of the sequence quality threshold enhances sample diversity through the inclusion of an increasing number of gSTs with ≥10 reads (e.g. 348 gSTs for the *P* = 0.015 test dataset; 20074 gSTs for the *P* = 0.05 dataset). However the identification of additional low abundance gSTs, considered as noise, after application of the EC model only provides a minor contribution to community structure where the proportions of the most abundant gSTs remain relatively unchanged.

Currently, the *gnd* database generated in this study contains 240 separate gSTs that differ from a consensus sequence by between 3 and 62 SNPs. MiSeq platform sequencing errors confounded clustering to group gSTs at the 99.6% similarity level (one SNP). The EC model however identifies gSTs by including a specified error based on the respective relative abundance of parent and daughter gSTs and removes gSTs where their relative abundance is assigned a probability of being generated in error. The advantage of using a DNA based method targetting a universal gene to type *E*. *coli* is that *gnd* sequence information from isolates that are untypable or rough using conventional serological methods can also be included in the database which is able to be expanded, modified or customised to include further *gnd* sequence information as more *E*. *coli* genome sequences are made available^30^. Although *Shigella* and *E*. *coli* belong to the same species complex^16, 17^, and share O-AGC^29^ for the purposes of this cattle study, *Shigella gnd* sequences were omitted from the database.

Our data suggest a significant host role in defining the overall bovine *E*. *coli* microbiota. Extraction method was a less significant factor influencing *E*. *coli* diversity and suggests that simple, boiled lysates, prepared from an RAMS enrichment are adequate for use as DNA templates in this amplicon sequencing method. There was also no evidence to suggest an impact of a daily administration with the two bifidobacterial strains on *E*. *coli* diversity. Whether housing the animals inside a dedicated research facility in a controlled environment may have impacted *E*. *coli* community structure through reduced transmission of environmentally acquired *E*. *coli* is unknown. Further experiments will examine the community structure of *E*. *coli* populations in animals reared under conventional New Zealand conditions.

In conclusion, this study has utilised culture-independent methods together with a model that overcomes the inherent similarity of target sequences to provide preliminary information on *E*. *coli* diversity within and between animals. Culture-independent methods to examine microbial communities can provide increased resolution of diversity not easily achievable with culture-based studies and their molecular characterisation. This metabarcoding amplicon sequencing method offers enhanced opportunities to study *E*. *coli* diversity across individuals of contrasting health status, to investigate temporal changes in *E*. *coli* community structure during maturation of the gut, or to assess the impact of stresses due to disease, antibiotics, or parturition. Furthermore the application of this method may provide insights demonstrating the complex nature of interralationships between different components of the *E*. *coli* microbiota. We have demonstrated the presence of many different *E*. *coli* types at low numbers which may constitute a pool responsible for the temporal variation and emergence of new *E*. *coli* types influenced by changes in animal health, age, diet or environmental conditions. The development of the *gnd* database may also provide a system through which a preliminary indication of *E*. *coli* serogroup can be attained without need for sera, with confirmation through serogroup specific PCR analysis^48^.

## Methods

### *gnd* alignment and PCR amplification

The *GND*-F and *GND*-R oligonucleotide primers^39^ were used to amplify a 710 bp region of the *gnd* locus from *E*. *coli*. Publicly available *E*. *coli* sequence data was obtained from GenBank, IMG and PATRIC^49^ databases and the *gnd* gene identified by name, EC designation (EC 1.1.1.44), or sequence. An alignment of a 642 bp (co-ordinates 382 to 1023 of 1407 bp *gnd* locus from *E*. *coli* O157:H7 EDL933, accession AE005174, gene Z3191, co-ordinates 2842138–2843544) region of the *gnd* locus encompassing the amino acids from the C-terminal domain of the NAD^+^ binding protein domain and the N-terminal domain of the phosphogluconate dehydrogenase domain was generated using the MUSCLE alignment feature of Geneious (v. 8.1.5). Degenerate oligonucleotide primers were designed from the 642 bp alignment described above using the Primer Design feature of Geneious (v. 8.1.5) and synthesised (Integrated DNA Technologies, Singapore): 2*gnd*F 5′-TCYATYATGCCWGGYGGVCAGAAAGAAG (*gnd* coordinates 415 to 442) and 2*gnd*R 5′-CATCAACCARGTAKTTACCSTCTTCATC (*gnd* coordinates 754 to 726) to generate a 340 bp amplicon. PCR protocols are included in the Supplementary Information.

### Animal sample processing and enrichment

The use of animals, including welfare, husbandry, experimental procedures, and the collection of samples used for this study, was approved by the Grasslands Animal Ethics Committee (Animal Ethics application 13518, Grasslands, Palmerston North, New Zealand) and performed in accordance with approved institutional and regulatory guidelines. Faecal and recto-anal mucosal swab (RAMS) samples were obtained from 23 calves aged 15 to 16 days old as part of an experiment to investigate the role of two bifidobacterial strains, *Bifidobacterium longum* AGR2137 and *Bifidobacterium pseudolongum* AGR2145^50^ on animal growth and performance. Probiotic strains were prepared fresh daily as described previously^50^ and orally dosed (approximately 10^10^ colony forming units per animal per day) to 12 (10 male, 2 female) individually penned calves that were housed in a separate room from 12 (10 male, 2 female) control animals. One calf from the bifidobacterial treatment group was euthanised due to pneumonia. RAMS swabs (n = 23) were added aseptically to 10 ml modified tryptone soya broth (mTSB, Fort Richard, Auckland, New Zealand) and vortexed briefly.

Four separate sample DNA extractions (designated pre-enrichment, ‘pre’; post-enrichment boiled lysate, ‘pob’; post-enrichment, spin-column, ‘por’ and faecal, ‘fec’) were prepared as sources of template DNA for amplicon sequencing. A pre-enrichment RAMS sample was obtained by removing 1 ml of mTSB prior to incubation at 42 °C for 18 hours. Post enrichment, duplicate 1 ml samples were removed for further processing. DNA extraction of the pre-enrichment RAMS sample and one of the two post-enrichment enrichment RAMS samples was performed using the High Pure PCR Template Preparation Kit (Roche, Auckland, New Zealand). A boiled lysate preparation was performed on the second post-enrichment RAMS sub-sample by centrifugation of the broth at 12,225 × g on a bench-top centrifuge and washing of the cell pellet in phosphate buffered saline (PBS, 0.01 M, pH 7.3). After another round of centrifugation, the cell pellet was fully resuspended in 1 ml of sterile MilliQ water and placed in a heating block at 100 °C for 10 min. DNA was extracted from fresh faecal samples (200 mg ± 10 mg) using a QIAamp Stool kit (Qiagen, Auckland, New Zealand) according to the manufacturer’s instructions. DNA extractions were stored at −20 °C until required. Stocks of the mTSB post-enrichment RAMS samples were made in glycerol (30% v/v) and stored at −80 °C.

### Bacterial isolation from enrichment cultures and *gnd* characterisation

Bacteria were resuscitated from −80 °C stocks by plating frozen enrichment culture on MacConkey agar plates (Fort Richard, Auckland, New Zealand) and incubating overnight at 37 °C. Up to seven well-spaced colonies with contrasting colours/morphologies were subcultured and the sequence of the 284 bp *gnd* amplicon from each isolate determined using Sanger sequencing as described above. *E*. *coli* and *Klebsiella* spp. were differentiated using specific culture media for citrate utilisation (Simmons citrate agar) and urease production (Fort Richard, Auckland, New Zealand). The confirmation of the serogroup of each isolate was attempted using serogroup-specific primers as described previously^40^.

### Library preparation and high throughput sequencing

Ninety two separate libraries were prepared from the DNA extracts obtained from each calf RAMS and faecal sample. The primers used for PCR were designed to generate custom amplicon duel index products amenable for MiSeq sequencing. Index sets C and D were used in the indexing with the primers following the standard Illumina MiSeq convention. The amplification protocol previously described was modified slightly whereby the annealing temperature was adjusted to 63 °C. Amplicons were purified (QIAquick PCR purification kit, Qiagen, Auckland, New Zealand) and quantified using a fluorometer (Qubit, Thermo Fisher Scientific, Auckland, New Zealand). Each amplicon library was diluted to approximately 5 nM and then pooled before Illumina MiSeq (V2) 2 × 250 bp paired end analysis was performed (New Zealand Genomics Limited, Massey Genome Service, Massey University, New Zealand). Four mock control libraries were prepared containing equimolar or contrasting concentrations of *gnd* amplicons from DNA extractions of serotyped *E*. *coli* strains. The standard PhiX loading control was added to the loading library at 10% due to this being a low-complexity custom amplicon library.

### Post sequencing read processing and gST enumeration

After standard on-machine MiSeq demultiplexing of the sequence reads into their constituent libraries, the sequences were analysed using a suite of tools for sequence quality control. For each library this involved the removal of any PhiX loading control through a mapping to the PhiX genome using the mapper BWA (http://bio-bwa.sourceforge.net/; version 0.7.12). The resulting SAM files were converted to fastQ files using the SamToFastq.jar program from the Picard suite (http://broadinstitute.github.io/picard/). These fastQ files were used as input for any adaptor removal using the “fastq-mcf” program from the ea-utils suite of tools (https://expressionanalysis.github.io/ea-utils/). Next, the libraries were run through a pair of quality control tools (FastQC (http://www.bioinformatics.babraham.ac.uk/projects/fastqc/; version 0.11.3) and SolexaQA++^51^ (http://solexaqa.sourceforge.net/; version 3.1.3) to assess the sequence quality, and to provide an overview of the total output reads. Finally an analysis with FastqScreen (http://www.bioinformatics.babraham.ac.uk/projects/fastq_screen/; version 0.4) was performed to check primarily for the presence of vector sequences, Illumina adapters and the PhiX loading control. Post sequencing, reads of >150 bp were filtered using sequencing quality thresholds of *P* = 0.05 (Phred score of Q13) to *P* = 0.001 (Phred score of Q30) prior to assembly of contiguous 284 bp sequences with FLASH^52^ (http://ccb.jhu.edu/software/FLASH/). Assembled sequences were mapped to the *gnd* database with unmatched sequences assigned md5 identifiers and unique gST classifications.

### Development and application of the Error Correction (EC) model to account for sequence processing errors

As there were many low abundance daughter gSTs that had not previously been observed, and that differed by just one base from a more highly abundant, previously observed parent sequence, it was theorised that these may have arisen due to sequencing error. An Error Correction (EC) model (Supplementary Note) was developed, where it was assumed that each observed sequence could have arisen through errors occurring at each base which would result in a difference at that base. It was assumed that the possible errors at each base position were equally likely and were independent, that at most one error occurs at each base, and that all ‘true’ sequences had been observed in the sample (in addition to ‘false’ sequences). To help satisfy the last of these assumptions, all libraries were pooled for this analysis. The model then allowed estimation of the error rate, the true abundance of each gST across the pooled libraries, as well as the likely contribution to the observed abundances from those true gSTs where they are the result of errors. The gSTs where the true abundance was zero likely arose due to error, and their observed abundances were then assigned to the parent sequences from which they were likely to have arisen.

### gST clustering and comparative analysis

A multidimensional scaling (MDS) plot was produced to demonstrate how gST abundances of the 80 sample libraries differed both within and between animals across extraction method (‘pob’, ‘por’, ‘pre’, ‘fec’). Multivariate analysis of variance was used to assess what proportion of the variation in relative gST abundances across the 80 sample libraries was due to the extraction method and between and within-calf variability. Extraction method was included as a fixed effect, and calf as a random effect, with the model being fit using PERMANOVA in Primer v6^53, 54^.

The genetic similarity of all 348 gSTs obtained from the 80 sample libraries was assessed by defining the distance between pairs of gSTs as the number of bases that differed. Agglomerative hierarchical clustering using complete linkage was then used to visualise how the gSTs were clustered. All 348 gSTs were ordered according to relative abundance of reads and CD-HIT^55, 56^ (http://weizhongli-lab.org/cd-hit/) used to cluster gSTs at the 99.6% identity level.

To illustrate the effect of the EC model, a minimum spanning tree was used to visualise the genetic diversity and abundances of the uncorrected and corrected data using the R package igraph^57^, where distances between gSTs were again measured by the number of different bases. In addition, heat maps allowed visualisation of how relative gST abundance differed across animals and methods.

### Code availability

All figures and analyses, with the exception of the PERMANOVA analysis, was performed in R version 3.3, and all R code is available in the Supplemental Material. Full details of the EC statistical model, and R code to fit the model, may be found in the Supplemental Material and https://github.com/mEpiLab/gnd_supplementary.

### Data availability

The novel *gnd* sequences reported in this study generated using culture and culture-independent methods have been deposited in GenBank/EMBL/DDBJ database (accession nos KX810237-KX810322 and KX894808-KX894899). Further sequence data is available from BioProject PRJNA353042 (BioSamples SAMN06010607 to SAMN06010702) in the form of respective read01 and read02 fastq files associated with each of the 96 libraries.

## Electronic supplementary material

Supplementary Info

Supplementary Table 2

Supplementary Table 3

Supplementary Table 6

Supplementary Table 7

Supplementary Table 8

Supplementary Table 9

Supplementary Table 10
