# Supplementary material for: Culture independent analysis using gnd as a target gene to assess Escherichia coli diversity and community structure
Source: Sci Rep. 2017 Apr 12;7:841. doi: 10.1038/s41598-017-00890-6 (PMC5429811; doi:10.1038/s41598-017-00890-6)
Supplement: Supplementary file 1 — Supplementary Info [file 41598_2017_890_MOESM1_ESM.pdf]

Supplementary Data in support of manuscript SREP-16-46998A:

Culture independent analysis using *gnd* as a target gene to assess *Escherichia coli* diversity and community structure.

Adrian L. Cookson[1,2*], Patrick J. Biggs[2,3], Jonathan C. Marshall[2,4], Angela Reynolds[1], Rose M. Collis[1], Nigel P. French[2], and Gale Brightwell[1].

[1] AgResearch Limited, Hopkirk Research Institute, Palmerston North, New Zealand

[2] mEpiLab, Hopkirk Research Institute, Massey University, Palmerston North, New Zealand

[3] Massey Genome Service, New Zealand Genomics Limited, Massey University, Palmerston North, New Zealand

[4] Institute of Fundamental Sciences, Massey University, Palmerston North, New Zealand

[*] Author for correspondence: Dr Adrian Cookson, Food Assurance & Meat Quality, AgResearch Limited, Hopkirk Research Institute, Private Bag 11008, Palmerston North 4442, New Zealand (Email: adrian.cookson@agresearch.co.nz)

Short running head: *E. coli* diversity and community structure

Read error model

Given that many of the unknown sequences have very low abundance and are very similar, being often only a single base pair different to known sequences that have high abundance in the sample, we assume that these sequences may have arisen due to read error.

Thus, we assume the observed sequences may be partitioned into true sequences where no read errors have occurred, and sequences where one or more errors have occurred. Let e be the probability that a sequence has a read error at a base, assumed to be constant across all bases. Further, assume that the probability that a sequence has a read error at two or more bases are independent, so that the probability of a sequence having errors at d distinct bases is e^d. Lastly, assume that we have observed all possible 'correct' sequences in the data, so that all observed sequences are either correct, or are generated via the above error process from one of the other observed sequences. Note that we assume that at most one error occurs at each base for a given sequence: We cannot tell whether multiple errors within the sequencing process occurred at a single base, only that it is in error.

Under this model, the probability of observing a sequence S_i may be conditioned over the potential sequences that generated it, $\{S_j\}$

$$P(S_i) = \sum_j P(S_i|S_j)P(S_j) = \sum_j e^{d_{ij}}(1-e)^{D-d_{ij}}p_j$$

where d_{ij} is the number of base pairs that differ between S_i and S_j, D is the number of bases, and p_j is the true prevalence of sequence j. Thus, the number Y_i of reads we observe (with or without error) of each sequence follows a Multinomial distribution with parameters N, the total number of reads observed, and $P(S_i)$ for each sequence i. The likelihood of the data are then

$$P(Y|e,p) = \prod_{i=1}^{N} \left[\sum_j e^{d_{ij}}(1-e)^{D-d_{ij}}p_j \right]^{Y_i}$$

As we are interested in the true number for each sequence (i.e. the unobserved data before read errors) we introduce latent variables x_{ij} as the number of reads of the true sequence j that contribute to the possibly modified sequence i, so that $Y_i = \sum_j x_{ij}$, with $\sum_i x_{ij}$ giving the true number for each sequence (possibly zero). Expanding the term with the power in the likelihood to introduce the latent variables gives

$$P(Y|e,p) = \prod_{i=1}^{N} \sum_{\{\sum_k x_{ik}=Y_i\}} \binom{Y_i}{x_{i1},\ldots,x_{iK}} \prod_j \left[e^{d_{ij}}(1-e)^{D-d_{ij}}p_j\right]^{x_{ij}}$$

where the summation is taken over all sets of latent variables that provide the observed read counts.

We fit the model, estimating e, p and X in a Bayesian context by conditioning the likelihood on the (unknown) values of the latent variables to give

$$P(Y|e,p,x) = \prod_{i=1}^{N} \binom{Y_i}{x_{i1},\ldots,x_{iK}} \prod_j \left[e^{d_{ij}}(1-e)^{D-d_{ij}}p_j\right]^{x_{ij}}$$

Assuming conjugate priors $p \sim \mathsf{Dirichlet}(\alpha)$ and $e \sim \mathsf{Beta}(\alpha_e, \beta_e)$ yields conditional posteriors of p and e that are also Dirichlet and Beta distributions:

$$P(p|Y,e,X) = \mathsf{Dirichlet}(\sum_i x_{ij} + \alpha)$$

$$P(e|Y,p,X) = \mathsf{Beta}(\sum_i \sum_j d_{ij}x_{ij} + \alpha_e, \sum_i \sum_j (D-d_{ij})x_{ij} + beta_e)$$

with the conditional posterior for the latent variables x being multinomial,

$$P(x_i|Y, e, p) = \mathsf{Multinomial}(Y_i, (q_{i1}, \ldots, q_{iK})),$$

where $q_{ij} = e^{d_{ij}}(1-e)^{D-d_{ij}}p_j$.

Thus, we can sample from the posterior using Monte Carlo Markov Chains (MCMC) with Gibbs sampling by sampling x followed by p and then e repeatedly.

Four chains of 51000 iterations sampling every 100th iteration after the first 1000 yields 2000 posterior samples for the parameters. Convergence of the chains were assessed by examining trace plots and calculating effective sample size and split $\hat{R}$ across chains.

The diagonal entries x_{jj} of x represent the number of reads of sequence j that are error-free, and thus $\frac{x_{jj}}{\sum_i x_{ji}}$ represents the fraction of the observed reads of sequence j that are error-free. We use a cut-off of 0.5 for this proportion, so that we assume sequence j has arisen due to read error if the proportion of genuine reads is less than 0.5. Experimenting with this cut-off value and find that the allocation of sequences to genuine or error is insensitive to this cut-off value, with a similar number of error-free sequences being found for a wide range of cut-off values.

All computer code for the MCMC algorithm and all other analyses are available in the supplementary material.

PCR reaction conditions.

PCR amplification of the 340 base pair 2*gnd*F/2*gnd*R fragment was undertaken using a mastermix containing 12.5ul 2 x KAPA HiFi Ready Mix (KAPA Biosystems, Wilmington, MA, USA) 10µl water, 0.75µl 2*gnd*F primer (10µM stock) and 0.75µl 2*gnd*R primer (10µM stock). Boiled lysates from serotyped *E. coli* were used as PCR reaction DNA templates (1µl) where a single well-spaced *E. coli* colony was emulsified in 400µl MilliQ water, boiled for 10minutes at 100°C in a heating block and then stored at -20°C until required. Amplicons were generated on a T100™ Thermal Cycler (Bio-Rad, Auckland, New Zealand) with a single denaturing step at 95°C for 5 minutes followed by 30 cycles of 98°C for 20 seconds, 60°C for 30 seconds and 72°C for 30 seconds. A final elongation step of 72°C for 5 minutes was followed by a 12°C hold step. Amplicons were visualised on a 2% (w/v) agarose gel stained with RedSafe (Custom Science, Auckland, New Zealand) stain using UV illumination. Amplicons were purified (QIAquick PCR purification kit, Qiagen, bio-strategy, Auckland, New Zealand) and quantified using spectrophotometry (NanoDrop, ThermoFisher Scientific, Auckland, New Zealand).

The amplification protocol previously described was modified slightly for the amplification of duel index products amenable for MiSeq sequencing whereby the annealing temperature was adjusted to 63°C.

Comparative analysis of *gnd* mock control libraries to establish test dataset for pipeline development.

Post sequencing, the proportion of assembled reads corresponding to the defined gSTs included in the four mock libraries with base quality scores exceeding a quality threshold of between 1 base call error every 20 nucleotides ($P = 0.05$ or Phred quality score of Q13), and 1 base call error every 1000 nucleotides ($P = 0.001$ or Phred quality score of Q30) (Supplementary Fig. 1) was determined. The respective read numbers provided an indication of MiSeq read bias between the most and least abundant gST from libraries where *gnd* amplicons were included at equimolar concentrations (Supplementary Table 2).

Compared to the four mock library datasets generated with the most stringent quality threshold of $P = 0.001$ (Phred quality score of Q30) with 4038 combined reads, twice as many mock library reads (8306) were generated with the $P = 0.015$ (Phred quality score of Q18.2) threshold with only a marginal reduction in total

reads matching component gSTs (*P* = 0.015, 98.92% and *P* = 0.01, 99.26%) (Supplementary Table 2). Thus the MiSeq dataset with a read quality threshold of 1 base call error every 67 nucleotides (*P* = 0.015) was chosen as a test dataset for the development of a framework for the analysis of *E. coli* diversity.

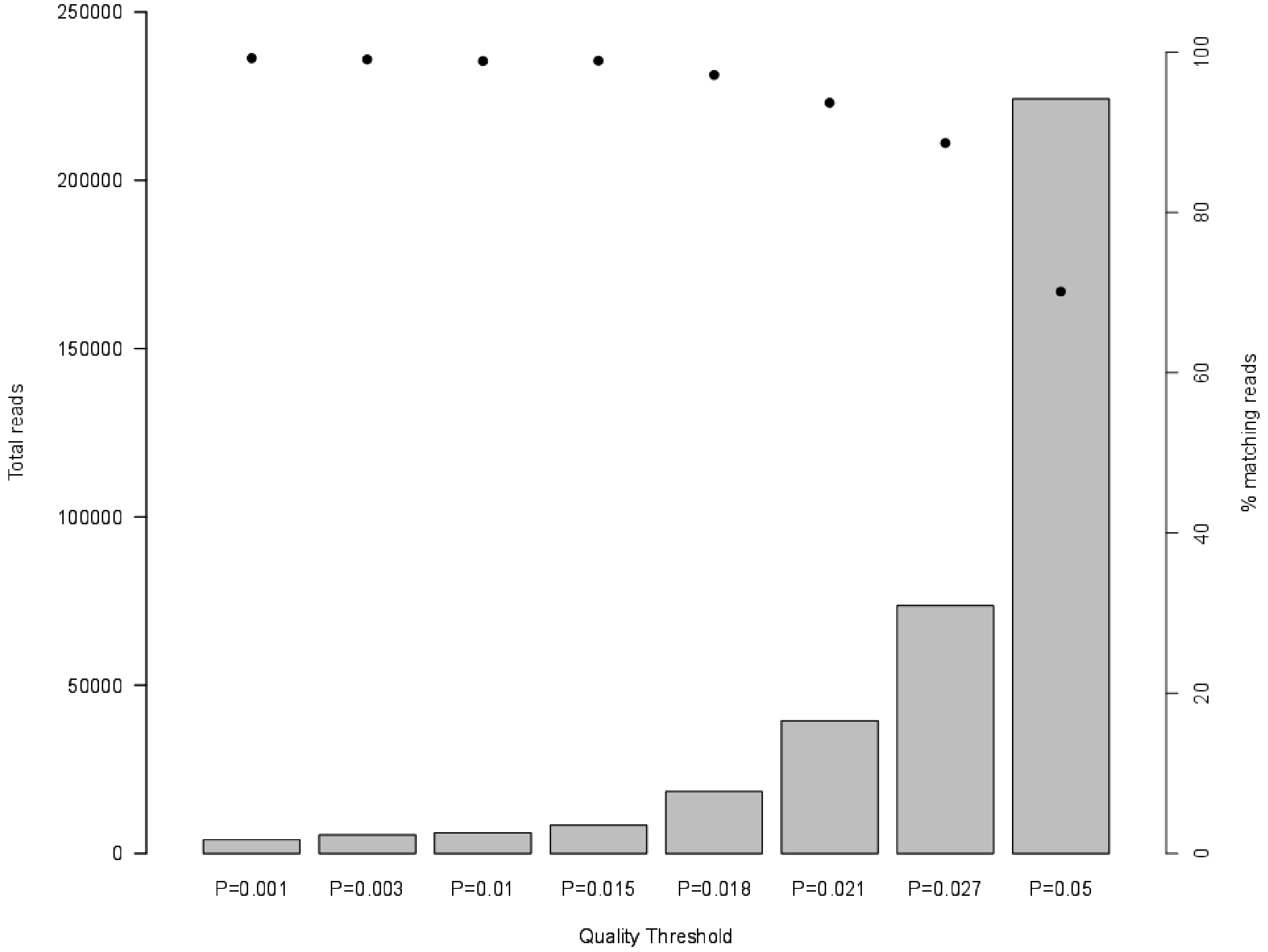

Supplementary Fig. 1 | Mock library analysis. To establish the sensitivity and specificity of the Illumina MiSeq platform for *gnd* amplicon sequence analysis, four mock control libraries were prepared containing equimolar or contrasting concentrations of up to seven different *gnd* amplicons. Amplicons were generated from DNA extractions of *E. coli* O26, O45, O103, O111, O121, O145, and O157 strains. Post sequence reads of > 150 bp were filtered using sequencing quality thresholds of *P* = 0.05 (Phred score of Q13) to *P* = 0.001 (Phred score of Q30) prior to assembly of contiguous 284 bp sequences. The total number of combined reads (primary y axis) from the four mock control libraries (bars) using each sequence quality threshold (x axis) is also illustrated against the percentage (secondary y axis) of assembled mock library reads matching the input *E. coli* O26, O45, O103, O111, O121, O145, and O157 *gnd* sequence types (•).

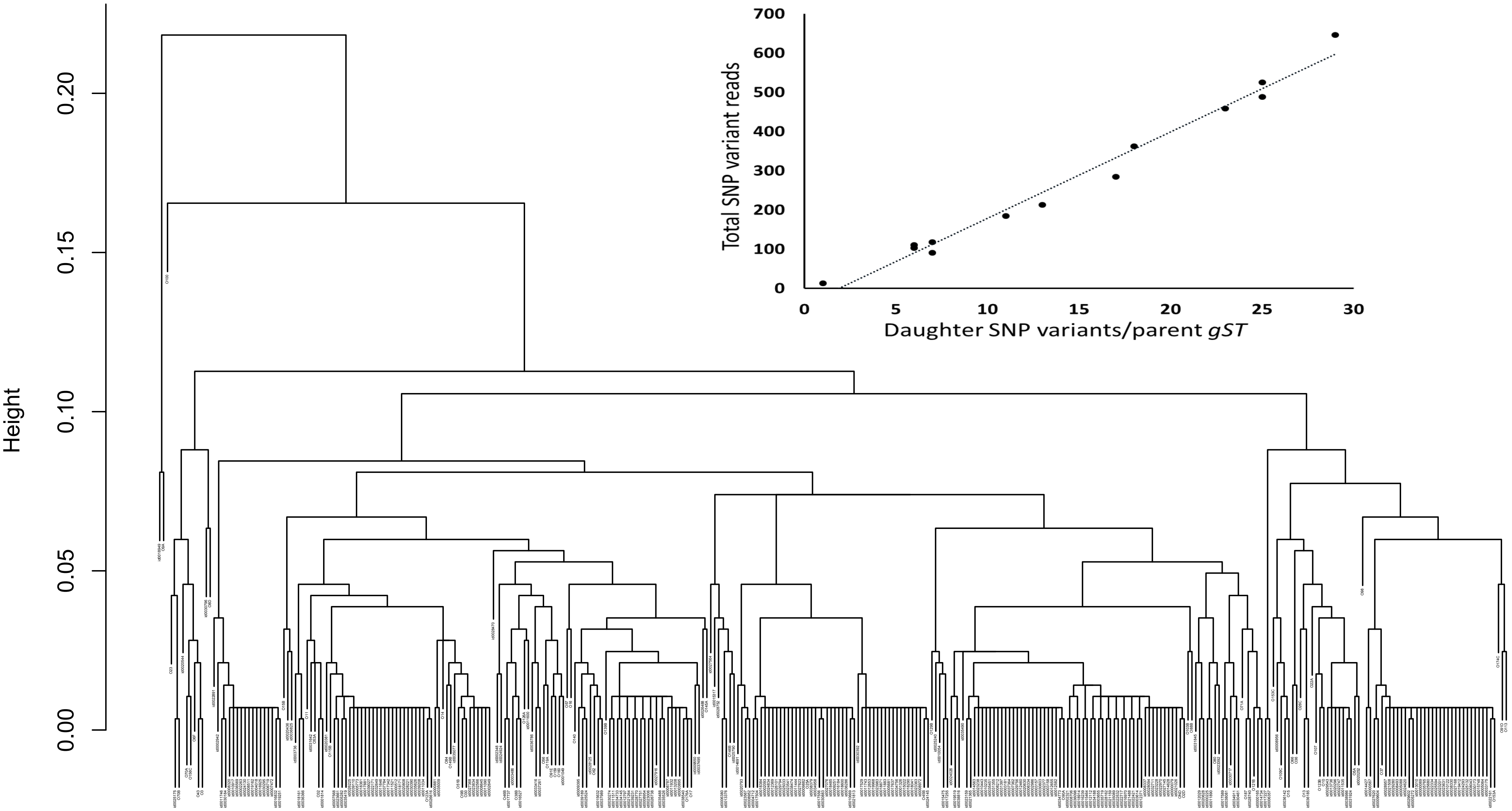
Height
0.00
0.05
0.10
0.15
0.20
Total SNP variant reads
0
100
200
300
400
500
600
700
0
5
10
15
20
25
30
Daughter SNP variants/parent *gST*

Supplementary Fig. 2 | Hierarchical clustering of *gnd* sequence types. Main figure. Clustering of 348 distinct *gnd* sequence types from amplicon libraries generated from template DNA prepared using four extraction methods (pre-enrichment, ‘pre’; post-enrichment, boiled lysate, ‘pob’; post-enrichment, spin-column, ‘por’ and faecal, ‘fec’) for each of 20 animals (i.e., 80 libraries) using a quality threshold of $P = 0.015$. Many clusters consist of a high abundance parent gST (e.g. O153A) that cluster with less abundant novel daughter gSTs that differed from the parent by one SNP suggesting that they were caused by sequencing error. Inset. The combined read total of the low abundance daughter gSTs was plotted against the read total of the respective high abundance parent gST and generated a broadly linear relationship providing further evidence of daughter gSTs generated by sequencing error.

a.

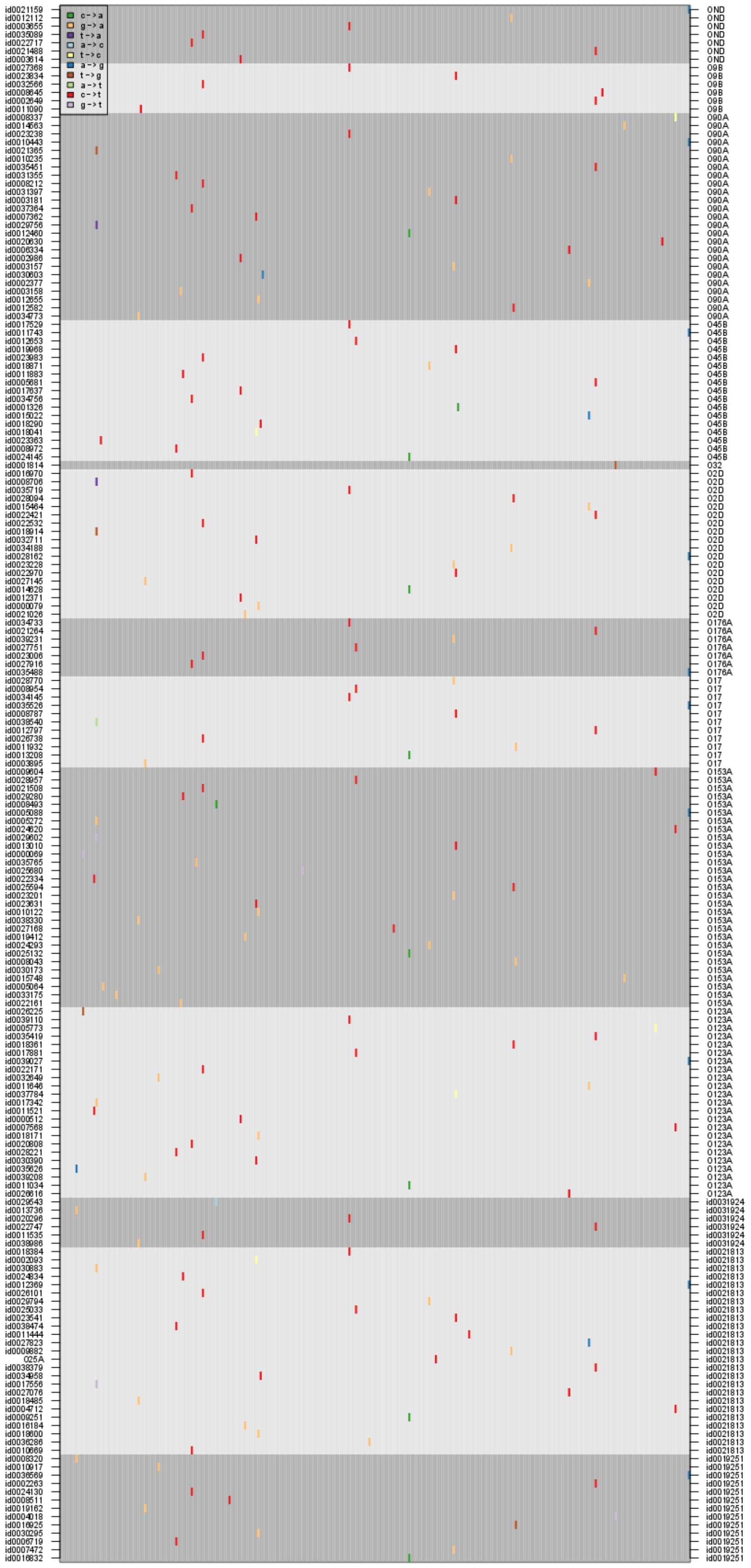

b.

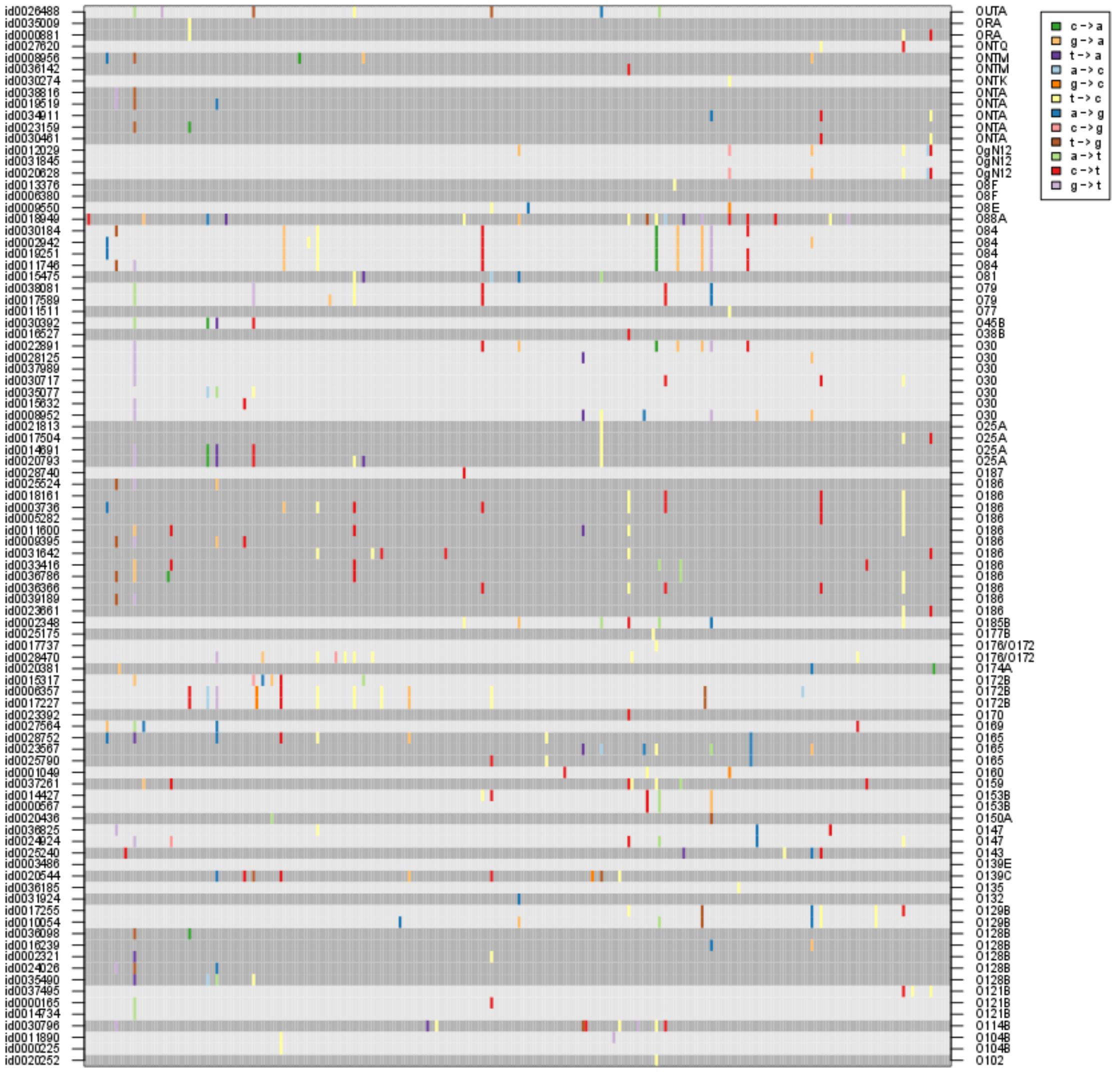

Supplementary Fig. 3 | SNP location within *gnd* sequence type (gST) amplicons. 348 *gnd* sequence types were identified from amplicon libraries generated from template DNA prepared using four extraction methods for each of 20 animals (i.e., 80 libraries) using a quality threshold of P = 0.015. **a.** Using the EC model, 188 gSTs (left) were identified as being likely associated with sequencing error due to low abundance. The *gnd* amplicon (284 bp) and associated SNP position (coloured) of each gST is mapped against probable source gST (right). SNP positions were found across the 284 bp *gnd* amplicon. **b.** The 92 novel gSTs (left) not matching the *gnd* sequence database were identified from sequence data using the EC model and SNPs mapped against closest matching gST from database (right). Novel gSTs differed from the closest matching gST from database by as many as 11 SNPs (id0017277 and O172B). Three novel gSTs matched gSTs from the

database (id0006380 and O8F, id0031845 and OgN12, and id0003486 and O139E); O8F, OgN12 and O139E gSTs were added to the database after preliminary analysis of the *P* = 0.015 dataset.

a.

2gndF/2gndR amplicon translated amino acid position

sample	probable_ source	1	2	3	4	5	6	7	8	9	10	11	12	13	14	15	16	17	18	19	20	21	22	23	24	25	26	27	28	29	30	31	32	33	34	35	36	37	38	39	40	41	42	43	44	45	46	47	48	49	50	51	52	53	54	55	56	57	58	59	60	61	62	63	64	65	66	67	68	69	70	71	72	73	74	75	76	77	78	79	80	81	82	83	84	85	86	87	88	89	90	91	92	93	94
id0007472	id0019251																																																										GD																																			
id0030295	id0019251																													AT																																																															
id0019162	id0019251												AT																																																																																
id0024130	id0019251																			PS																																																																									
id0010917	id0019251														AT																																																																														
id0010669	id0021813																			PS																																																																									
id0036286	id0021813																																													MI																																															
id0018600	id0021813																													AT																																																															
id0016184	id0021813																											DN																																																																	
id0018485	id0021813											AT																																																																																	
id0034958	id0021813																													AV																																																															
id0009882	id0021813																																																																			AT																									
id0038986	id0031924											AT																																																																																	
id0039208	O123A												AT																																																																																
id0020808	O123A																			PS																																																																									
id0018171	O123A																													AT																																																															
id0000512	O123A																										AV																																																																		
id0011521	O123A				AV																																																																																								
id0032649	O123A														AT																																																																														
id0018361	O123A																																																																			AV																									
id0022161	O153A																	GD																																																																											
id0015748	O153A																																																																																				DN								
id0030173	O153A														AT																																																																														
id0019412	O153A																											DN																																																																	
id0027168	O153A																																																	AV																																											
id0038330	O153A											AT																																																																																	
id0010122	O153A																													AT																																																															
id0023201	O153A																																																										GD																																			
id0025594	O153A																																																																			AV																									
id0022334	O153A				AV																																																																																								
id0025680	O153A																																			MI																																																									
id0003895	O17												AT																																																																																
id0028770	O17																																																										GD																																			
id0027916	O176A																			PS																																																																									
id0039231	O176A																																																										GD																																			
id0021026	O2D																											DN																																																																	
id0000079	O2D																													AT																																																															
id0012371	O2D																										AV																																																																		
id0027145	O2D												AT																																																																																
id0023228	O2D																																																										GD																																			
id0034188	O2D																																																																			AT																									
id0028094	O2D																																																																			AV																									
id0016970	O2D																			PS																																																																									
id0023363	O45B					PL																																																																																							
id0018290	O45B																													AV																																																															
id0001326	O45B																																																											LM																																	
id0034756	O45B																			PS																																																																									
id0017637	O45B																										AV																																																																		
id0034773	O90A											AT																																																																																	
id0012582	O90A																																																																			AV																									
id0012655	O90A																													AT																																																															
id0003158	O90A																	GD																																																																											
id0003157	O90A																																																										GD																																			
id0002986	O90A																										AV																																																																		
id0037364	O90A																			PS																																																																									
id0010235	O90A																																																																			AT																									
id0014563	O90A																																																																																				DN								
id0011090	O9B											AV																																																																																	
id0003614	OND																										AV																																																																		
id0022717	OND																			PS																																																																									
id0012112	OND																																																																			AT																									

b.

2gndF/2gndR amplicon translated amino acid position

sample	closest_match	1	2	3	4	5	6	7	8	9	10	11	12	13	14	15	16	17	18	19	20	21	22	23	24	25	26	27	28	29	30
id0011890	O104B																														
id0010054	O129B																														
id0025240	O143																														
id0024924	O147																														
id0000567	O153B																														
id0014427	O153B																														
id0037261	O159																														
id0025790	O165																														
id0023567	O165																														
id0028752	O165																														
id0017227	O172B																			EQ											
id0006357	O172B																			EQ											
id0020381	O174A				VI																										
id0025175	O177B																														
id0002348	O185B																														
id0036786	O186									TN																					
id0033416	O186																														
id0003736	O186																						VI								
id0008952	O30																														
id0022891	O30																														
id0017589	O79																											AT			
id0011746	O84																						VI								
id0019251	O84																						VI								
id0002942	O84																						VI								
id0030184	O84																						VI								
id0018949	O88A																														
id0020628	OgN12																														
id0012029	OgN12																														
id0026488	OUTA																														

sample	closest_match	31	32	33	34	35	36	37	38	39	40	41	42	43	44	45	46	47	48	49	50	51	52	53	54	55	56	57	58	59	60
id0011890	O104B																												GC		
id0010054	O129B																														
id0025240	O143																														
id0024924	O147																														
id0000567	O153B																														
id0014427	O153B																														
id0037261	O159																														
id0025790	O165																														
id0023567	O165																														
id0028752	O165																														
id0017227	O172B																														
id0006357	O172B																														
id0020381	O174A																														
id0025175	O177B																														
id0002348	O185B																														
id0036786	O186																														
id0033416	O186																														
id0003736	O186																														
id0008952	O30																														
id0022891	O30																														
id0017589	O79																														
id0011746	O84																														
id0019251	O84																														
id0002942	O84																														
id0030184	O84																														
id0018949	O88A																														
id0020628	OgN12																														
id0012029	OgN12																														
id0026488	OUTA																														

sample	closest_match	61	62	63	64	65	66	67	68	69	70	71	72	73	74	75	76	77	78	79	80	81	82	83	84	85	86	87	88	89	90	91	92	93	94
id0011890	O104B																																		
id0010054	O129B			TS																															
id0025240	O143					DE																													
id0024924	O147			TS																															
id0000567	O153B			TS																															
id0014427	O153B			TS																															
id0037261	O159					EV																													
id0025790	O165													KE																					
id0023567	O165	NS												KE																					
id0028752	O165													KE																					
id0017227	O172B								SA																										
id0006357	O172B								SA										ED																
id0020381	O174A																																	QK	
id0025175	O177B		FS																																
id0002348	O185B			TS																															
id0036786	O186					EV																													
id0033416	O186			TS		EV																													
id0003736	O186																																		
id0008952	O30	NS																																	
id0022891	O30					EK																													
id0017589	O79																																		
id0011746	O84					EK																													
id0019251	O84					EK																													
id0002942	O84					EK																													
id0030184	O84					EK																													
id0018949	O88A					DE																													
id0020628	OgN12																																NT		
id0012029	OgN12																																NT		
id0026488	OUTA			TS																															

Supplementary Fig. 4 | Impact of *gnd* SNPs on resultant 94 amino acid sequence translation. 348 *gnd* sequence types were identified from amplicon libraries generated from template DNA prepared using four extraction methods for each of 20 animals (i.e., 80 libraries) using a quality threshold of $P = 0.015$. **a.** Using the EC model, 188 gSTs were identified as being likely associated with sequencing error due to low abundance. Upon translation, 61 of 188 gSTs (left) likely associated with sequencing error were associated with non-synonymous amino acid changes. gST and likely source gST illustrated on y axis. Position of non-synonymous amino acid changes along 94 amino acid chain is illustrated on the x axis with first amino acid abbreviation representing the original amino acid, and second amino acid abbreviation representing the non-synonymous change. **b.** A total of 92 novel gSTs not matching the *gnd* sequence database were identified from sequence data using the EC model (left) and were associated with 40 non-synonymous amino acid changes. gST and closest matching gST from database illustrated on y axis. Position of non-synonymous amino acid changes along 94 amino acid chain is illustrated on the x axis with first amino acid abbreviation representing the original amino acid, and second amino acid abbreviation representing the non-synonymous change. In total 22 of 40 non-synonymous amino acid changes were associated with amino acid positions 61 to 68.

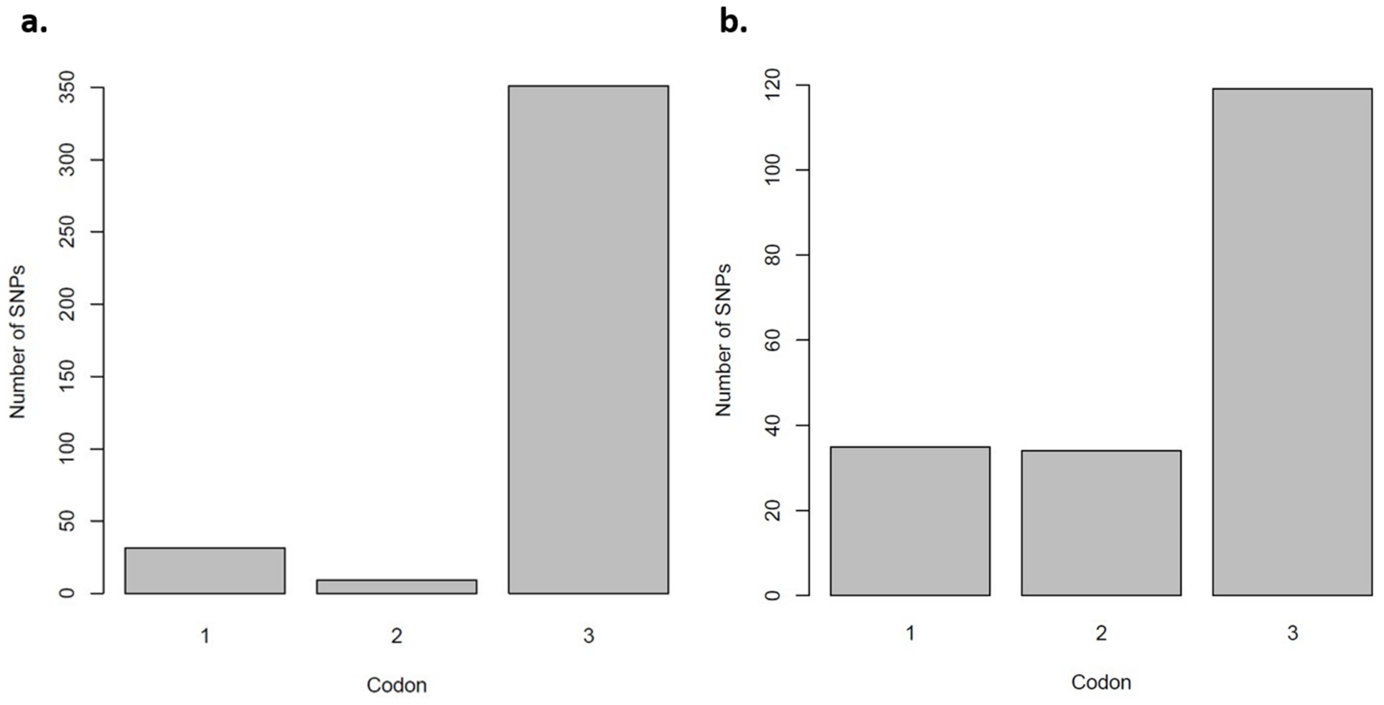

Supplementary Fig. 5 | SNP and codon position. 348 *gnd* sequence types were identified from amplicon libraries generated from template DNA prepared using four extraction methods for each of 20 animals (i.e., 80 libraries) using a quality threshold of $P = 0.015$. **a.** A total of 92 novel gSTs not matching the *gnd* sequence database were identified from sequence data using the EC model. For the 92 novel gSTs, the total number of SNPs at each codon position, compared to closest matching gST from database is displayed. gSTs contained up to 11 SNPs **b.** Using the EC model, 188 gSTs, each with one SNP compared to the parent gST, were identified as being likely associated with sequencing error due to low abundance. Codon analysis indicated that SNPs occurring at variable codon positions 1 and 2 for novel gSTs (a.) were less frequent than gSTs associated with sequencing error (b.) (χ^2 test: $P < 0.001$) reflecting the biological burden and selection pressures associated with amino acid changes at codon positions 1 and 2. SNPs occurring at codon position 3 are more likely to be associated with synonymous substitutions that do not alter amino acid sequences.

Supplementary Table 1 | Publicly–available and novel sequence data were used to generate a *gnd* database containing representative sequences from all 184 recognised *E. coli* O serogroups, 28 *gnd* sequence types (gSTs) from *E. coli* described as untypable or 'rough', and three new, as yet undesignated O serogroups. Sequence accession numbers or references provided where necessary.

gST	**Serogroup/serotype**	**Strain**	**Accession/reference**	**Matching *gST***
O1A	O1:H6	LR09	JDVF00000000	O7B
O1B	O1:H20		[1]	
O2A	O2:H4	1.2741	AEZI00000000	
O2B	O2:H4	03-2815	DQ472566	O50
O2C	O2:NM	9.1649	AEZY00000000	
O2D	O2:H29		[1]	O128B
O3	O3	U14-41	EU694097	O21
O4A	O4:H43	ECOR67	M64327	O8B
O4B	O4:H5	03-3266	DQ472567	
O5/O71	O5/O71	R394	KX810316	
O5A	O5:H4	ATCC23502	CAPL00000000	O71B ORB
O5B	O5:NM	03-2825	DQ472555	
O6A	O6:H16	B2C	AUZS00000000	
O6B	O6:H1	03-2638	DQ472568	
O6C	O6:H1	ECOR56	M63829	
O6D	O6:H10	ECOR10	M63821	
O6E	O6:H10	ECOR11	M63822	
O6F	O6:H49	ATCC9637	CP002185	
O7A	O7:NM	CE10	CP003034	
O7B	O7:NT	RS76	CP013048	O1A
O8A	O8:H16	03-2639	DQ472569	
O8B	O8:H49	5344-1	LHCP00000000	O4A
O8C	O8	93404-41	AB811598	
O8D	O8	IAI1	CU928160	
O8E	O8:H19	MHI813	AFDZ00000000	
O8F	O8:H19	KCJ852	LSFT00000000	
O9A	O9	HS	CP000802	
O9B	O9:H51	AGR609	KX810292	
O10	O10:H4	ATCC23506	CAPK00000000	ONTN
O11	O11:NM	PCN033	CP006632	
O12	O12	Bi626	AB811600	
O13	O13	Su4321	AB972413	O135
O14	O14	Su4411	AB972414	
O15A	O15	F7902-41	AY647261	O49B ONTM
O15B	O15:H18	K1516	JHJE00000000	
O16	O16	F11119-41	AB811601	O150B
O17	O17:H18	UMN026	CU928163	O44 O73B O77
O18A	O18ac	D-M3219-54	AB811603	
O18B	O18ab	F10018-41	AB811602	
O19A	O19	F8188-41	AB811604	O148
O19B	O19:H34	SMS-3-5	CP000970	

O20	O20	P7a	AB811605	O116B O137 O22B
O21	O21		EU694098	O3
O22A	O22:H8		KM822859	O156B
O22B	O22	E14a	AB811606	O116B O137 O20
O23	O23	E39a	AB811607	
O24	O24	E41a	DQ220292	
O25A	O25:NM	E2539c	JHJV00000000	
O25B	O25b:H4	EC958	HG941718	
O25C	O25:H1	03-2637	DQ472570	
O25D	O25	E47a	GU014554	
O26A	O26:H6	clone 3	AY973404	
O26B	O26:H11	2011C-3655	JHLN00000000	O158 ONTO
O27	O27	F9884-41	GU014555	
O28AB	O28ab	K1a	AB811608	
O28AC	O28ac	Kattwijk	AB811609	O42
O29	O29	1085-87	EU294173	
O30	O30	P2a	AB811610	
O32	O32:H37	P4	AJQW00000000	
O33	O33	E40	AB811611	
O34	O34	H304	AB811612	
O35	O35	E77a	FJ940774	
O36	O36	H502a	AB811613	
O37	O37:NM	AGR194	KX810250	
O38A	O38	F11621-41	AB811615	
O38B	O38:H21		[1]	
O39	O39:NM	F8704-2	JHHJ00000000	
O40	O40	H710	EU296417	
O41	O41	H710C	AB811617	
O42	O42	P11a	AB811618	O28AC
O43	O43	Bi7455-41	AB811619	
O44	O44:H18	042	NC_017626	O17 O73B O77
O45A	O45:H7	S88	CU463050	ONTF
O45B	O45:H2	01-3147	JHOA00000000	
O46A	O46:H38		[1]	O134B
O46B	O46	P1c	AB811621	O170/O172 O173 O176/O172
O48	O48	U8-41	AB811622	
O49A	O49	U12-41	AB811623	
O49B	O49:H10	AGR014	KX810239	O15A ONTM
O50	O50:H4	U18-41	KX810319	O2B
O51A	O51	U19-41	AB812020	
O51B	O51:NM		[1]	
O52	O52	U20-41	AY528413	
O53A	O53	Bi-7327	AB812021	
O53B	O53	S6966	JICI00000000	O55B
O54	O54	Su3972-41	AB812085	
O55A	O55:H7	05-0376	DQ472575	O121C O89A
O55B	O55	S7438	JICF00000000	O53B
O55C	O55:H6	ICC219	AB353133	
O56	O56	Su3684-41	DQ220293	
O57	O57	F8198-41	AB972415	O64
O58	O58	F8962-41	EU294175	

O59	O59	F9095-41	AY654590	
O60	O60	F10167a-41	AB812022	
O61	O61	F10167a-42	GU220362	
O62	O62	F10524-41	AB812023	O171 O172A O181 O68A
O63	O63	F10598-41	AB812024	
O64	O64	K6b	AB812025	O57
O65	O65	K11a	AB812026	
O66	O66	P1a	KX810312	
O68A	O68:H4	P7d	KX810313	O171 O172A O181 O62
O68B	O68:H24	245G	KX810237	
O68C	O68:NM	ERL10-0195	KX810306	O177A ONTB
O69	O69	P9b	AB812028	ONTI
O70A	O70	P9c	AB812029	
O70B	O70:H11	AGR007	KX810238	O177B O91A
O71A	O71	P10a	GU445927	
O71B	O71:HR	T39G	KX810317	O5A ORB
O73A	O73	P12a	DQ000313	
O73B	O73:H18		[1]	O17 O44 O77
O74A	O74	E3a	AB812030	
O74B	O74:H42	4588-1	LGZW00000000	
O75	O75:H8	AGR831	KX810296	
O76A	O76:NM	AGR183	KX810247	ONTG
O76B	O76:H19		[1]	
O77	O77	E10	AB972416	O17 O44 O73B
O78	O78:H12	00-3279	JFBE00000000	O79
O79	O79:H7	06-3501	JHNM00000000	O78
O80	O80	E71	AB812032	
O81	O81	ED1a	NC_011745	
O82	O82	H14	AB812034	O172B
O83	O83	H17a	AB812035	
O84	O84:H2	AGR081	KX810244	
O85	O85:H1	03-3638	DQ472571	
O86A	O86:H43	ECOR23	M63826	
O86B	O86	H35	AY220982	
O87	O87:H12	H40	KX810308	
O88A	O88:H25	97.0264	AEZP00000000	
O88B	O88	H53	AB812037	
O89A	O89	ECOR20	M63824	O121C O55A
O89B	O89	H68	AB812038	
O90A	O90	H77	AB812039	O109A O117A O127 ONTA
O90B	O90:H8	AGR047	KX810242	ONTD
O91A	O91:NM	AGR414	KX810286	O177B O70B
O91B	O91:NM	AGR945	KX810300	
O91C	O91:H21	B2F1	AGTI00000000	O96
O92	O92	H308a	AB812040	
O93	O93	2885-2	AB812041	O175 OUTB
O95	O95	H311a	AB812042	
O96	O96	H319	AB812043	O91C
O97	O97	H320a	AB812044	
O98	O98:NM	AGR151	KX810245	
O99	O99	H504c	FJ940773	

O100	O100	H509a	AB812045	
O101A	O101	H510a	AB812046	O162
O101B	O101	B41	AFAH00000000	
O102	O102	H511	AB812047	
O103A	O103:H11	04-3023	JHOD00000000	O184
O103B	O103:H25	AGR566	KX810290	
O104A	O104	H519	AF361371	O112AC
O104B	O104:H4	2011C-3493	CP003289	
O105	O105:H8	H520b	KX810309	
O106A	O106	H521a	AB972417	
O106B	O106		DQ000315	
O107	O107:H8	W114	KX810322	O117B
O108	O108	H708b	AB812048	O131B
O109A	O109	H709c	HM485572	O117A O127 O90A ONTA
O109B	O109:NM	AGR226a	KX810253	
O109C	O109	12662-2	LDCZ00000000	ONTL
O110	O110	H711c	AB812049	
O111	O111:NM	Stoke-W	AF078736	ONTE
O112AB	O112ab	32W	EU296413	
O112AC	O112ac	Guanabara	AB812050	O104A
O113	O113:H21	93-0016	DQ472561	
O114A	O114	26w	AY573377	
O114B	O114	S6685	JICA00000000	
O115A	O115:NM	AGR698	KX810293	
O115B	O115:H18	03-3645	DQ472572	O183 OUTC
O116A	O116	28w	AB812051	
O116B	O116:H49	7712-3	LHCM00000000	O137 O20 O22B
O117A	O117:H25	02-0714	DQ472577	O109A O127 O90A ONTA
O117B	O117:H7	05-0379	DQ472576	O107
O118	O118	31w	AB972418	O151
O119A	O119:H25		[1]	O182 ONTH
O119B	O119:H4	03-3458	JHNV00000000	
O120	O120	35w	AB812052	
O121A	O121	F5/B	KX810307	
O121B	O121:H7	2009C-3299	JHHC00000000	
O121C	O121	ECOR21	M63825	O55A O89A
O123A	O123	43w	AB972419	O186
O123B	O123:H45	NT526	KX810310	
O124	O124	Ew227	AB972420	O144B O164
O125	O125	Ew2129	AB812053	
O126A	O126:H27	D92-09	LEKZ00000000	
O126B	O126:H6	W1118	KX810321	
O126C	O126	S7005	JICG00000000	O132
O127	O127:H6	E2348/69	FM180568	O109A O117A O90A ONTA
O128A	O128:H21	R175	KX810314	
O128B	O128:H21	DEC14A	AIHK00000000	O2D
O128C	O128:H2	DEC11A	AIGV00000000	ORA
O128D	O128:H7	DEC13A	AIHF00000000	
O129A	O129		EU296424	
O129B	O129:NM	AGR187	KX810248	
O130A	O130		EU296421	

O130B	O130:H38	492-1	LDDI00000000	
O131A	O131	S239	AB812055	
O131B	O131:H25	AGR053	KX810243	O108
O132	O132	N87	AB812056	O126C
O133	O133	N282	AB812057	
O134A	O134	4370-53	AB812058	O185B
O134B	O134:H38	1223-4	LHCT00000000	O46A
O135	O135		EU296423	O13
O136	O136	1111-55	AB812059	
O137	O137	RVC1787	AB972423	O116B O20 O22B
O138	O138		DQ109551	
O139A	O139:H1		[1]	O170
O139B	O139:H19		[1]	
O139C	O139		DQ109552	
O139D	O139:H28	E24377A	CP000800	
O139E	O139:H1	S1191	AFEA00000000	
O140	O140	CDC149-51	AB812060	
O141	O141		DQ868765	
O142A	O142	C771	AB812061	
O142B	O142:H38	16118-2	LHCW00000000	
O142C	O142:NM		[1]	
O143	O143:H26	4608-58	JTCO00000000	
O144A	O144	1624-56	AB812062	
O144B	O144	53638	AAKB00000000	O124 O164
O145A	O145:NM	03-4699	DQ472563	
O145B	O145:H28	RM13516	CP006262	
O145C	O145:H46	AGR718	KX810295	O146A
O146A	O146	AGR036	KX810241	O145C
O146B	O146:H21	2010C-3325	JASR00000000	
O146C	O146:H28		[1]	
O146D	O146	ATCC8739	CP000946	
O147	O147	G1253	DQ868766	
O148	O148	E519-66	DQ167407	O19A
O149A	O149:H10		DQ091854	
O149B	O149:NM	DEC7B	AIGB00000000	O157C O166
O150A	O150	1935	EU294168	
O150B	O150:H6	SE15	AP009378	O16
O150C	O150:H8	AGR704	KX810294	
O151	O151	880-67	AB972424	O118
O152A	O152:H38	Trh48	KX810318	
O152B	O152:H28	SE11	AP009240	
O153A	O153	3.3884	AFAC00000000	O156A ONTQ
O153B	O153:H2	2010C-5034	JHMH00000000	
O153C	O153	14097	AB812063	O178 ONTP
O154A	O154:H31		[1]	
O154B	O154	E1541-68	AB812064	
O155	O155		AY657020	
O156A	O156	AGR567	KX810291	O153A ONTQ
O156B	O156	E1585-68	AB812065	O22A
O156C	O156:NM		[1]	
O157A	O157:H16	13A81	AF176364	

O157B	O157:H45	EC95-42	AB602249	
O157C	O157:H12	G5933	AF176363	O149B O166
O157D	O157:H7	EDL933	AE005174	
O158	O158:H11	AGR024	KX810240	O26B ONTO
O159	O159	E2476-72	EU294176	
O160	O160	E110-69	AB812066	
O161	O161	G1254	GU220361	
O162	O162	10B-1	AB812067	O101A
O163	O163	SN3B-1	AB812068	
O164	O164	SC647	AB972425	O124 O144B
O165A	O165:H25	2012C-4227	CP013029	
O165B	O165:H25	2012C-4227	CP013029	
O166	O166	G1216	GU299794	O149B O157C
O167	O167	E10702	EU296408	
O168	O168	E10710	EU296403	
O169	O169	1792-54	AB812069	
O170	O170	745-56	AB812070	O139A
O170/O172	O170/O172:H49	R227	KX810315	O173 O176/O172 O46B
O171	O171	244-54	AB812071	O172A O181 O62 O68A
O172A	O172:H21		[1]	O171 O181 O62 O68A
O172B	O172	G1092	AY545992	O82
O172C	O172:NM	AGR197	KX810251	ONTJ
O173	O173:NM	CCUG36541	KX810305	O170/O172 O176/O172 O46B
O174A	O174	C8/55	AB812072	
O174B	O174:H46	I-151	AQGO00000000	
O174C	O174:H8	02-07607	AQGN00000000	
O175	O175	C12/55	AB812073	O93 OUTB
O176	O176	E29518	AB812074	ONTK
O176/O172	O176/O172:H49	W1108	KX810320	O170/O172 O173 O46B
O177A	O177:NM	03-3974	DQ472573C	O68C ONTB
O177B	O177:H11	AGR413	KX810285	O70B O91A
O178	O178	E54071	AB812075	O153C ONTP
O179	O179	E43478	AB812076	
O180	O180	86-381	AB812077	
O181	O181	92-1250	AB812078	O171 O172A O62 O68A
O182	O182	99-1287	AB812079	O119A ONTH
O183	O183:H18	5710-2	LHCQ0000000000	O115B OUTC
O184	O184	99-4473	AB812080	O103A
O185A	O185	99-6301	AB812081	
O185B	O185:H7	6842-1	LHCO00000000	O134A
O186	O186	182-02	AB812082	O123A
O187	O187	559-59	AB812083	
OgN10	OgN10	090823	LC125930	
OgN12	OgN12	110062	LC125931	
OgN8	OgN8	102755	LC125928	
OMA	OM:H18	ECOR47	M63828	
ONA	ON:NM	ECOR45	M64326	
ONB	ON:H10	ECOR65	M64331	
ONC	ON:NM	ECOR68	M64330	
OND	ON:NM	ECOR69	M64328	
ONTA	ONT:H10	AGR256	KX810255	O109A O117A O127 O90A

ONTB	ONT:HNT	AGR372	KX810283	O68C O177A
ONTC	ONT:HNT	ECC-1470	CP010344	
ONTD	ONT:NM	AGR159	KX810246	O90B
ONTE	ONT	S7380	JICD00000000	O111
ONTF	ONT:NM	AGR362	KX810282	O45A
ONTG	ONT:NM	AGR236	KX810254	O76A
ONTH	ONT:H25	AGR188	KX810249	O119A O182
ONTI	ONT:H6	AGR492	KX810288	O69
ONTJ	ONT:NM	AGR536	KX810289	O172C
ONTK	ONT:NM	AGR423	KX810287	O176A
ONTL	ONT:NM	AGR259	KX810256	O109C
ONTM	ONT:H10	AGR333	KX810281	O15A O49B
ONTN	ONT:NM	AGR223	KX810252	O101A
ONTO	ONT:NM	AGR889	KX810298	O158 O26B
ONTP	ONT:H7	AGR908	KX810299	O153C O178
ONTQ	ONT:H25	1932	LHCR00000000	O153A O156A
ONTR	ONT:HNT	AA86	AFET00000000	
ORA	OR:H2	AGR844	KX810297	O128C
ORB	OR:NM	AGR375	KX810284	O5A O71B
OUTA	OUT:H16		[1]	
OUTB	OUT:H28		[1]	O93 O175
OUTC	OUT:H18	EHOUT32	AB627352	O115B O183
OUTD	OUT:H11		[1]	

[1] www.corefacility.ca/ecoli_typer/

Supplementary Table 4 | Multivariate analysis of variance was undertaken to assess the proportion of variation in gST abundances across libraries due to treatment, extraction method and between and within-calf variability. Extraction method was included as a fixed effect, and calf as a random effect, with the model being fit using PERMANOVA. Amplicon libraries (n=80) were generated from template DNA prepared using four extraction methods (pre-enrichment, 'pre'; post-enrichment, boiled lysate, 'pob'; post-enrichment, spin-column, 'por' and faecal, 'fec' for each of 20 animals.

PERMANOVA table of results

**Source**	**df**	**SS**	**MS**	**Pseudo-F**	**P(perm)**	**Unique perms**
Treatment	1	1.0791	1.0791	0.94641	0.5263	9336
Extraction	3	0.41554	0.13851	2.3557	0.0004	9902
Animal(Tr)	18	20.524	1.1402	19.392	0.0001	9733
Residual	57	3.3516	0.06			

**Groups**	**t**	**P(perm)**	**Unique perms**
por, pob	1.158	0.3682	9942
por, pre	1.3932	0.0766	9949
por, fec	1.4431	0.0269	9961
pob, pre	1.5417	0.0159	9935
pob, fec	1.4767	0.022	9945
pre, fec	1.8379	0.0433	9928

Supplementary Table 5 | Total number reads associated with individual gSTs identified using the EC model and CD-HIT (99.6% similarity) respectively.

gSTs present with the EC-method but absent with CD-Hit

The following are those gSTs retained with the error correction method but removed with CD-HIT.

**gST**	***abundance***	***closest* gST**	***closest_abund***	***closest_dist***
OND	6754	O2D	12373	1
O176A	6426	O17	9425	1
id0006380	1144	id0013376	2114	1
id0011511	114	O17	9425	1
O38B	84	id0016527	1655	1
O30	68	O9B	7350	1
id0036185	58	O13	173	1
id0017737	52	O46B	408	1
O3	45	O43	268	1
id0014734	27	O121B	2306	1
id0017589	26	id0038081	31	1
id0019519	24	id0038816	53	1
id0017227	24	id0006357	1164	1
id0025175	20	O70B	677	1
id0034911	16	id0030461	36	1
id0009395	16	id0025524	17	1
id0015632	14	id0037989	19	1
id0015475	14	id0017255	347	1
id0011746	12	id0030184	18	1
id0039189	12	id0026225	45	1
O187	11	id0028740	15	1
id0036142	11	O15	156	1
id0023567	10	id0020793	14	1
id0018161	10	id0036366	14	1
id0020628	10	id0012029	15	1
id0011890	10	id0000225	48	1

They are legitimate due to:

1. High abundance (so unlikely to be errors)

2. Low abundance, and the closest serogroup is not abundant enough to produce this many errors (given the estimated error rate).

gSTs present with the CD-hit method but absent with the EC-method.

The following are those gSTs retained with CD-HIT but removed with the error correction method.

gST	*abundance*	*closest* gST	*closest_abund*	*closest_dist*
id0034733	29	O176A	6426	1
id0021159	27	OND	6754	1
id0003655	20	OND	6754	1
id0021264	19	O176A	6426	1
id0022717	16	OND	6754	1
id0035089	16	OND	6754	1
id0027751	15	O176A	6426	1
id0021488	14	OND	6754	1
id0012112	14	OND	6754	1
id0035488	12	O176A	6426	1
id0027916	12	O176A	6426	1
id0039231	12	O176A	6426	1
id0003614	11	OND	6754	1
id0023006	11	O176A	6426	1

These are universally low abundance, and close to a serogroup with high abundance.

All gSTs found in *P* = 0.015 dataset at a relative abundance of ≥ 10 reads.

gST	*abundance*	*closest* gST	*closest_abund*	*closest_dist*	*EC_method*	*CD_hit*
O153A	17014	id0009604	57	1	TRUE	TRUE
O90A	16528	id0008337	80	1	TRUE	TRUE
O123A	13818	id0026225	45	1	TRUE	TRUE
id0021813	13191	O25A	46	1	TRUE	TRUE
O2D	12373	OND	6754	1	TRUE	TRUE
O45B	11552	id0017529	38	1	TRUE	TRUE
O17	9425	O176A	6426	1	TRUE	TRUE
id0019251	9091	id0008320	45	1	TRUE	TRUE
O9B	7350	O30	68	1	TRUE	TRUE
OND	6754	O2D	12373	1	TRUE	FALSE
O176A	6426	O17	9425	1	TRUE	FALSE
id0031924	4231	id0013736	38	1	TRUE	TRUE
O109C	2520	O15	156	2	TRUE	TRUE
O121B	2306	id0014734	27	1	TRUE	TRUE
id0024924	2293	id0002348	11	5	TRUE	TRUE
O32	2187	id0001814	13	1	TRUE	TRUE
id0013376	2114	id0006380	1144	1	TRUE	TRUE
id0016527	1655	O38B	84	1	TRUE	TRUE
id0006357	1164	id0017227	24	1	TRUE	TRUE
id0006380	1144	id0013376	2114	1	TRUE	FALSE
O57	938	O149B	640	7	TRUE	TRUE
O5A	934	O123B	92	3	TRUE	TRUE
O6F	754	O156C	59	4	TRUE	TRUE

O70B	677	id0025175	20	1	TRUE	TRUE
O15B	643	O30	68	8	TRUE	TRUE
O149B	640	O84	138	4	TRUE	TRUE
O160	637	id0001049	18	3	TRUE	TRUE
O16	562	id0011511	114	6	TRUE	TRUE
O70A	517	O90A	16528	3	TRUE	TRUE
O113	498	O91D	439	7	TRUE	TRUE
O91D	439	O113	498	7	TRUE	TRUE
O46B	408	id0017737	52	1	TRUE	TRUE
id0017255	347	id0015475	14	1	TRUE	TRUE
id0035009	310	id0000881	10	2	TRUE	TRUE
O9A	298	id0018949	26	23	TRUE	TRUE
O43	268	O3	45	1	TRUE	TRUE
O26C	265	O123B	92	5	TRUE	TRUE
O11	249	id0031642	57	6	TRUE	TRUE
O159	245	O91E	170	3	TRUE	TRUE
O62	202	O51B	73	2	TRUE	TRUE
O14	182	id0035009	310	6	TRUE	TRUE
O13	173	id0036185	58	1	TRUE	TRUE
O91E	170	O159	245	3	TRUE	TRUE
O15	156	id0036142	11	1	TRUE	TRUE
O84	138	id0017881	34	3	TRUE	TRUE
O37	121	O153A	17014	2	TRUE	TRUE
O107	115	O123B	92	5	TRUE	TRUE
id0011511	114	O17	9425	1	TRUE	FALSE
O36	109	O66	30	4	TRUE	TRUE
O71A	93	O149B	640	4	TRUE	TRUE
O123B	92	id0020252	62	2	TRUE	TRUE
O119A	84	O56	12	4	TRUE	TRUE
O38B	84	id0016527	1655	1	TRUE	FALSE
id0008337	80	O90A	16528	1	FALSE	FALSE
id0027564	79	O146B	26	7	TRUE	TRUE
O153C	77	O15	156	8	TRUE	TRUE
id0031845	76	O2D	12373	4	TRUE	TRUE
O51B	73	O30	68	1	TRUE	TRUE
id0037261	70	id0033416	26	2	TRUE	TRUE
id0036786	69	id0026225	45	5	TRUE	TRUE
O30	68	O9B	7350	1	TRUE	FALSE
O103B	67	O43	268	6	TRUE	TRUE
id0020252	62	O123B	92	2	TRUE	TRUE
id0030274	60	O176A	6426	1	TRUE	TRUE
O156C	59	O70A	517	3	TRUE	TRUE
id0036185	58	O13	173	1	TRUE	FALSE
id0031642	57	id0037784	21	5	TRUE	TRUE
id0009604	57	O153A	17014	1	FALSE	FALSE
id0028957	56	O153A	17014	1	FALSE	FALSE
id0038816	53	id0021365	25	1	TRUE	TRUE
id0017737	52	O46B	408	1	TRUE	FALSE
O98	51	id0014427	25	14	TRUE	TRUE
id0000225	48	id0011890	10	1	TRUE	TRUE
id0036825	48	O84	138	4	TRUE	TRUE

id0023392	47	O160	637	4	TRUE	TRUE
O25A	46	id0021813	13191	1	FALSE	FALSE
id0008706	46	O2D	12373	1	FALSE	FALSE
O3	45	O43	268	1	TRUE	FALSE
id0008320	45	id0019251	9091	1	FALSE	FALSE
id0030717	45	id0038081	31	2	TRUE	TRUE
id0026225	45	O123A	13818	1	FALSE	FALSE
id0035719	44	O2D	12373	1	FALSE	FALSE
O115B	42	id0026225	45	2	TRUE	TRUE
id0039110	42	O123A	13818	1	FALSE	FALSE
id0023238	41	O90A	16528	1	FALSE	FALSE
id0024620	41	O153A	17014	1	FALSE	FALSE
id0029602	40	O153A	17014	1	FALSE	FALSE
O140	40	id0039189	12	3	TRUE	TRUE
id0029280	40	O153A	17014	1	FALSE	FALSE
id0021508	40	O153A	17014	1	FALSE	FALSE
id0028470	39	O46B	408	9	TRUE	TRUE
id0018384	39	id0021813	13191	1	FALSE	FALSE
O88B	38	id0002321	13	4	TRUE	TRUE
id0024834	38	id0021813	13191	1	FALSE	FALSE
id0013736	38	id0031924	4231	1	FALSE	FALSE
id0035419	38	O123A	13818	1	FALSE	FALSE
id0005272	38	O153A	17014	1	FALSE	FALSE
id0017529	38	O45B	11552	1	FALSE	FALSE
id0005088	38	O153A	17014	1	FALSE	FALSE
id0010443	37	O90A	16528	1	FALSE	FALSE
id0011600	37	id0037784	21	5	TRUE	TRUE
id0015317	36	O146B	26	6	TRUE	TRUE
id0030461	36	id0008337	80	1	TRUE	TRUE
O8D	34	id0003181	21	11	TRUE	TRUE
id0017881	34	O123A	13818	1	FALSE	FALSE
id0034145	33	O17	9425	1	FALSE	FALSE
id0026101	32	id0021813	13191	1	FALSE	FALSE
id0038081	31	id0017589	26	1	TRUE	TRUE
O66	30	O36	109	4	TRUE	TRUE
id0027368	30	O9B	7350	1	FALSE	FALSE
id0010054	29	id0015475	14	4	TRUE	TRUE
id0034733	29	O176A	6426	1	FALSE	TRUE
id0027620	29	id0009604	57	1	TRUE	TRUE
id0010235	29	O90A	16528	1	FALSE	FALSE
id0012369	29	id0021813	13191	1	FALSE	FALSE
id0013010	28	O153A	17014	1	FALSE	FALSE
id0037495	27	O121B	2306	3	TRUE	TRUE
id0031355	27	O90A	16528	1	FALSE	FALSE
id0022532	27	O2D	12373	1	FALSE	FALSE
id0021159	27	OND	6754	1	FALSE	TRUE
id0014734	27	O121B	2306	1	TRUE	FALSE
O146B	26	id0025790	12	5	TRUE	TRUE
id0036569	26	id0019251	9091	1	FALSE	FALSE
id0017589	26	id0038081	31	1	TRUE	FALSE
id0033416	26	id0037261	70	2	TRUE	TRUE

id0035451	26	O90A	16528	1	FALSE	FALSE
id0018949	26	O9A	298	23	TRUE	TRUE
id0029794	25	id0021813	13191	1	FALSE	FALSE
id0022171	25	O123A	13818	1	FALSE	FALSE
id0014427	25	id0000567	14	2	TRUE	TRUE
id0022421	25	O2D	12373	1	FALSE	FALSE
id0012653	25	O45B	11552	1	FALSE	FALSE
id0021365	25	O90A	16528	1	FALSE	FALSE
id0030883	25	id0021813	13191	1	FALSE	FALSE
id0019519	24	id0038816	53	1	TRUE	FALSE
id0017504	24	id0004712	10	1	TRUE	TRUE
id0017227	24	id0006357	1164	1	TRUE	FALSE
id0000069	24	O153A	17014	1	FALSE	FALSE
id0039027	24	O123A	13818	1	FALSE	FALSE
id0011743	24	O45B	11552	1	FALSE	FALSE
O174C	23	O10	14	9	TRUE	TRUE
id0036098	23	id0018914	17	1	TRUE	TRUE
id0035526	23	O17	9425	1	FALSE	FALSE
id0015464	23	O2D	12373	1	FALSE	FALSE
O145A	23	O30	68	6	TRUE	TRUE
id0031397	22	O90A	16528	1	FALSE	FALSE
id0002263	22	id0019251	9091	1	FALSE	FALSE
id0037364	21	O90A	16528	1	FALSE	FALSE
id0026488	21	O2D	12373	7	TRUE	TRUE
id0003181	21	O90A	16528	1	FALSE	FALSE
id0002942	21	id0019251	9091	3	TRUE	TRUE
id0037784	21	O123A	13818	1	FALSE	FALSE
id0023541	21	id0021813	13191	1	FALSE	FALSE
id0025033	21	id0021813	13191	1	FALSE	FALSE
id0032649	21	O123A	13818	1	FALSE	FALSE
id0019968	21	O45B	11552	1	FALSE	FALSE
id0032566	20	O9B	7350	1	FALSE	FALSE
id0028752	20	id0025790	12	7	TRUE	TRUE
id0025175	20	O70B	677	1	TRUE	FALSE
id0003655	20	OND	6754	1	FALSE	TRUE
id0038474	20	id0021813	13191	1	FALSE	FALSE
id0034188	20	O2D	12373	1	FALSE	FALSE
id0008212	20	O90A	16528	1	FALSE	FALSE
id0023983	20	O45B	11552	1	FALSE	FALSE
id0025240	20	id0015475	14	4	TRUE	TRUE
O100	19	O153C	77	35	TRUE	TRUE
id0037989	19	O30	68	1	TRUE	TRUE
id0023228	19	O2D	12373	1	FALSE	FALSE
id0021264	19	O176A	6426	1	FALSE	TRUE
id0020436	19	id0031642	57	6	TRUE	TRUE
id0008645	19	O9B	7350	1	FALSE	FALSE
id0038540	19	O17	9425	1	FALSE	FALSE
id0020544	19	id0015475	14	9	TRUE	TRUE
id0011444	19	id0021813	13191	1	FALSE	FALSE
id0022334	19	O153A	17014	1	FALSE	FALSE
O139B	18	id0011511	114	4	TRUE	TRUE

id0002649	18	O9B	7350	1	FALSE	FALSE
id0001049	18	O160	637	3	TRUE	TRUE
id0034756	18	O45B	11552	1	FALSE	FALSE
id0030184	18	id0008320	45	1	TRUE	TRUE
id0020296	18	id0031924	4231	1	FALSE	FALSE
id0038379	17	id0021813	13191	1	FALSE	FALSE
id0035626	17	O123A	13818	1	FALSE	FALSE
id0024130	17	id0019251	9091	1	FALSE	FALSE
id0005282	17	id0035419	38	1	TRUE	TRUE
id0027823	17	id0021813	13191	1	FALSE	FALSE
id0014691	17	id0020793	14	2	TRUE	TRUE
id0009550	17	id0011511	114	3	TRUE	TRUE
id0003486	17	O38B	84	2	TRUE	TRUE
id0007568	17	O123A	13818	1	FALSE	FALSE
id0028162	17	O2D	12373	1	FALSE	FALSE
id0025524	17	id0009395	16	1	TRUE	TRUE
id0005681	17	O45B	11552	1	FALSE	FALSE
id0023661	17	id0007568	17	1	TRUE	TRUE
id0018914	17	O2D	12373	1	FALSE	FALSE
id0025680	17	O153A	17014	1	FALSE	FALSE
id0016970	17	O2D	12373	1	FALSE	FALSE
id0034911	16	id0030461	36	1	TRUE	FALSE
id0026738	16	O17	9425	1	FALSE	FALSE
id0022717	16	OND	6754	1	FALSE	TRUE
id0020630	16	O90A	16528	1	FALSE	FALSE
id0012797	16	O17	9425	1	FALSE	FALSE
id0012460	16	O90A	16528	1	FALSE	FALSE
id0009395	16	id0025524	17	1	TRUE	FALSE
id0035089	16	OND	6754	1	FALSE	TRUE
id0022970	16	O2D	12373	1	FALSE	FALSE
O129B	16	id0017255	347	6	TRUE	TRUE
id0038330	16	O153A	17014	1	FALSE	FALSE
id0028221	16	O123A	13818	1	FALSE	FALSE
id0011521	16	O123A	13818	1	FALSE	FALSE
id0025594	16	O153A	17014	1	FALSE	FALSE
O80	16	id0023392	47	4	TRUE	TRUE
O23	16	O70A	517	9	TRUE	TRUE
O22A	16	O123B	92	8	TRUE	TRUE
id0027751	15	O176A	6426	1	FALSE	TRUE
id0024026	15	id0019519	24	2	TRUE	TRUE
id0023159	15	id0021365	25	1	TRUE	TRUE
id0013208	15	O17	9425	1	FALSE	FALSE
id0012029	15	id0020628	10	1	TRUE	TRUE
id0011932	15	O17	9425	1	FALSE	FALSE
id0030796	15	id0001814	13	10	TRUE	TRUE
id0027145	15	O2D	12373	1	FALSE	FALSE
id0009882	15	id0021813	13191	1	FALSE	FALSE
id0020808	15	O123A	13818	1	FALSE	FALSE
id0018171	15	O123A	13818	1	FALSE	FALSE
id0010122	15	O153A	17014	1	FALSE	FALSE
id0035765	15	O153A	17014	1	FALSE	FALSE

id0028740	15	O187	11	1	TRUE	TRUE
id0005773	15	O123A	13818	1	FALSE	FALSE
id0029543	15	id0031924	4231	1	FALSE	FALSE
id0021488	14	OND	6754	1	FALSE	TRUE
id0019162	14	id0019251	9091	1	FALSE	FALSE
id0012371	14	O2D	12373	1	FALSE	FALSE
id0003157	14	O90A	16528	1	FALSE	FALSE
id0002986	14	O90A	16528	1	FALSE	FALSE
id0017556	14	id0021813	13191	1	FALSE	FALSE
id0039208	14	O123A	13818	1	FALSE	FALSE
id0025132	14	O153A	17014	1	FALSE	FALSE
id0015632	14	id0037989	19	1	TRUE	FALSE
id0020793	14	id0023567	10	1	TRUE	TRUE
id0018041	14	O45B	11552	1	FALSE	FALSE
O10	14	O123B	92	2	TRUE	TRUE
id0030390	14	O123A	13818	1	FALSE	FALSE
id0007362	14	O90A	16528	1	FALSE	FALSE
id0015475	14	id0017255	347	1	TRUE	FALSE
id0000512	14	O123A	13818	1	FALSE	FALSE
id0012112	14	OND	6754	1	FALSE	TRUE
id0023201	14	O153A	17014	1	FALSE	FALSE
id0032711	14	O2D	12373	1	FALSE	FALSE
id0011883	14	O45B	11552	1	FALSE	FALSE
id0018871	14	O45B	11552	1	FALSE	FALSE
id0008954	14	O17	9425	1	FALSE	FALSE
id0028094	14	O2D	12373	1	FALSE	FALSE
id0000567	14	id0014427	25	2	TRUE	TRUE
id0036366	14	id0018161	10	1	TRUE	TRUE
O53A	13	id0001814	13	5	TRUE	TRUE
id0027076	13	id0021813	13191	1	FALSE	FALSE
id0022891	13	id0011746	12	4	TRUE	TRUE
id0003736	13	id0036366	14	4	TRUE	TRUE
id0002321	13	id0008706	46	1	TRUE	TRUE
id0008972	13	O45B	11552	1	FALSE	FALSE
id0008511	13	id0019251	9091	1	FALSE	FALSE
id0014628	13	O2D	12373	1	FALSE	FALSE
id0006334	13	O90A	16528	1	FALSE	FALSE
id0029756	13	O90A	16528	1	FALSE	FALSE
id0027168	13	O153A	17014	1	FALSE	FALSE
id0015022	13	O45B	11552	1	FALSE	FALSE
id0001814	13	O32	2187	1	FALSE	FALSE
id0017342	13	O123A	13818	1	FALSE	FALSE
id0010917	13	id0019251	9091	1	FALSE	FALSE
O56	12	O119A	84	4	TRUE	TRUE
id0035488	12	O176A	6426	1	FALSE	TRUE
id0027916	12	O176A	6426	1	FALSE	TRUE
id0025790	12	id0013376	2114	4	TRUE	TRUE
id0018485	12	id0021813	13191	1	FALSE	FALSE
id0011746	12	id0030184	18	1	TRUE	FALSE
id0008952	12	id0023567	10	1	TRUE	TRUE
id0000165	12	id0014734	27	1	TRUE	TRUE

id0030603	12	O90A	16528	1	FALSE	FALSE
id0011034	12	O123A	13818	1	FALSE	FALSE
id0035490	12	id0008706	46	3	TRUE	TRUE
id0039189	12	id0026225	45	1	TRUE	FALSE
id0023363	12	O45B	11552	1	FALSE	FALSE
id0039231	12	O176A	6426	1	FALSE	TRUE
id0034958	12	id0021813	13191	1	FALSE	FALSE
id0018290	12	O45B	11552	1	FALSE	FALSE
id0023631	12	O153A	17014	1	FALSE	FALSE
id0023834	12	O9B	7350	1	FALSE	FALSE
id0008787	12	O17	9425	1	FALSE	FALSE
id0002093	12	id0021813	13191	1	FALSE	FALSE
id0014563	12	O90A	16528	1	FALSE	FALSE
O187	11	id0028740	15	1	TRUE	FALSE
O126A	11	id0023661	17	6	TRUE	TRUE
id0036142	11	O15	156	1	TRUE	FALSE
id0030295	11	id0019251	9091	1	FALSE	FALSE
id0016925	11	id0019251	9091	1	FALSE	FALSE
id0016184	11	id0021813	13191	1	FALSE	FALSE
id0012655	11	O90A	16528	1	FALSE	FALSE
id0011090	11	O9B	7350	1	FALSE	FALSE
id0004018	11	id0019251	9091	1	FALSE	FALSE
id0003895	11	O17	9425	1	FALSE	FALSE
id0003614	11	OND	6754	1	FALSE	TRUE
id0002377	11	O90A	16528	1	FALSE	FALSE
id0000079	11	O2D	12373	1	FALSE	FALSE
id0033175	11	O153A	17014	1	FALSE	FALSE
id0023006	11	O176A	6426	1	FALSE	TRUE
id0016239	11	id0015464	23	1	TRUE	TRUE
id0011535	11	id0031924	4231	1	FALSE	FALSE
id0008043	11	O153A	17014	1	FALSE	FALSE
id0022747	11	id0031924	4231	1	FALSE	FALSE
id0030392	11	id0011883	14	3	TRUE	TRUE
id0020381	11	id0023661	17	3	TRUE	TRUE
id0002348	11	id0024924	2293	5	TRUE	TRUE
id0028125	11	id0037989	19	2	TRUE	TRUE
id0018361	11	O123A	13818	1	FALSE	FALSE
id0028770	11	O17	9425	1	FALSE	FALSE
id0008493	11	O153A	17014	1	FALSE	FALSE
id0035077	10	O30	68	3	TRUE	TRUE
id0034773	10	O90A	16528	1	FALSE	FALSE
id0022161	10	O153A	17014	1	FALSE	FALSE
id0021026	10	O2D	12373	1	FALSE	FALSE
id0016832	10	id0019251	9091	1	FALSE	FALSE
id0012582	10	O90A	16528	1	FALSE	FALSE
id0010669	10	id0021813	13191	1	FALSE	FALSE
id0008956	10	O109C	2520	5	TRUE	TRUE
id0007472	10	id0019251	9091	1	FALSE	FALSE
id0006719	10	id0019251	9091	1	FALSE	FALSE
id0000881	10	id0035009	310	2	TRUE	TRUE
id0036286	10	id0021813	13191	1	FALSE	FALSE

id0023567	10	id0020793	14	1	TRUE	FALSE
id0018600	10	id0021813	13191	1	FALSE	FALSE
id0009251	10	id0021813	13191	1	FALSE	FALSE
id0018161	10	id0036366	14	1	TRUE	FALSE
id0003158	10	O90A	16528	1	FALSE	FALSE
id0038986	10	id0031924	4231	1	FALSE	FALSE
id0004712	10	id0021813	13191	1	FALSE	FALSE
id0026616	10	O123A	13818	1	FALSE	FALSE
id0005064	10	O153A	17014	1	FALSE	FALSE
id0024145	10	O45B	11552	1	FALSE	FALSE
id0015748	10	O153A	17014	1	FALSE	FALSE
id0030173	10	O153A	17014	1	FALSE	FALSE
id0024293	10	O153A	17014	1	FALSE	FALSE
id0019412	10	O153A	17014	1	FALSE	FALSE
id0001326	10	O45B	11552	1	FALSE	FALSE
id0017637	10	O45B	11552	1	FALSE	FALSE
id0011646	10	O123A	13818	1	FALSE	FALSE
id0020628	10	id0012029	15	1	TRUE	FALSE
id0011890	10	id0000225	48	1	TRUE	FALSE
